# Autoantibody profiles in Alzheimer´s, Parkinson´s, and dementia with Lewy bodies: altered IgG affinity and IgG/IgM/IgA responses to alpha-synuclein, amyloid-beta, and tau in disease-specific pathological patterns

**DOI:** 10.1186/s12974-024-03293-3

**Published:** 2024-12-03

**Authors:** Luisa Knecht, Katrine Dalsbøl, Anja Hviid Simonsen, Falk Pilchner, Jean Alexander Ross, Kristian Winge, Lisette Salvesen, Sara Bech, Anne-Mette Hejl, Annemette Løkkegaard, Steen G Hasselbalch, Richard Dodel, Susana Aznar, Gunhild Waldemar, Tomasz Brudek, Jonas Folke

**Affiliations:** 1 Centre for Neuroscience and Stereology, Department of Neurology, Bispebjerg and Frederiksberg Hospital, Copenhagen University Hospital, Nielsine Nielsens Vej 6B, Entrance 11B, 2. floor, Copenhagen, NV DK-2400 Denmark; 2 Copenhagen Center for Translational Research, Bispebjerg and Frederiksberg Hospital, Copenhagen University Hospital, Nielsine Nielsens Vej 4B, Copenhagen, NV DK-2400 Denmark; 3 Danish Dementia Research Centre, Copenhagen University Hospital - Rigshospitalet, University of Copenhagen, Blegdamsvej 9, Copenhagen Ø, DK-2100 Denmark; 4 Chair of Geriatric Medicine, Center for Translational Neuro- and Behavioral Sciences, University Duisburg-Essen, Hufelandstraße 55, DE-45147 Essen, Germany; 5 Odense University Hospital, University of Southern Denmark, Copenhagen, Denmark; 6 Department of Neurology, Bispebjerg and Frederiksberg Hospital, Copenhagen University Hospital, Nielsine Nielsens Vej 7, Copenhagen, NV DK-2400 Denmark; 7 Department of Clinical Medicine, Faculty of Health and Medical Sciences, University of Copenhagen, Blegdamsvej 3B, Copenhagen Ø, DK-2100 Denmark

**Keywords:** Alzheimer’s disease, Dementia with lewy bodies, Parkinson’s disease, Naturally occurring autoantibodies, Alpha-synuclein, Amyloid-beta, Tau

## Abstract

**Background:**

Alzheimer’s disease (AD) and Parkinson’s disease (PD) are leading neurodegenerative disorders marked by protein aggregation, with AD featuring amyloid-beta (Aβ) and tau proteins, and PD alpha-synuclein (αSyn). Dementia with Lewy bodies (DLB) often presents with a mix of these pathologies. This study explores naturally occurring autoantibodies (nAbs), including Immunoglobulin (Ig)G, IgM, and IgA, which target αSyn, Aβ and tau to maintain homeostasis and were previously found altered in AD and PD patients, among others.

**Main text:**

We extended this investigation across AD, PD and DLB patients investigating both the affinities of IgGs and levels of IgGs, IgMs and IgAs towards αSyn, Aβ and tau utilizing chemiluminescence assays. We confirmed that AD and PD patients exhibited lower levels of high-affinity anti-Aβ and anti-αSyn IgGs, respectively, than healthy controls. AD patients also showed diminished levels of high-affinity anti-αSyn IgGs, while anti-tau IgG affinities did not differ significantly across groups. However, DLB patients exhibited increased anti-αSyn IgG but decreased anti-αSyn IgM levels compared to controls and PD patients, with AD patients showing a similar pattern. Interestingly, AD patients had higher anti-Aβ IgG but lower anti-Aβ IgA levels than DLB patients. DLB patients had reduced anti-Aβ IgM levels compared to controls, and anti-tau IgG levels were lower in AD than PD patients, who had reduced anti-tau IgM levels compared to controls. AD patients uniquely showed higher anti-tau IgA levels. Significant correlations were observed between clinical measures and nAbs, with negative correlations between anti-αSyn IgG affinity and levels in DLB patients and a positive correlation with anti-αSyn IgA levels in PD patients. Disease-specific changes in nAb levels and affinity correlations were identified, highlighting altered immune responses.

**Conclusion:**

This study reveals distinctive nAb profiles in AD, DLB, and PD, pinpointing specific immune deficiencies against pathological proteins. These insights into the autoreactive immune system’s role in neurodegeneration suggest nAbs as potential markers for vulnerability to protein aggregation, offering new avenues for understanding and possibly diagnosing these conditions.

**Supplementary Information:**

The online version contains supplementary material available at 10.1186/s12974-024-03293-3.

## Background

Neurodegenerative diseases are mainly characterized by the pathological accumulation of specific proteins, which play a pivotal role in disease progression. Alzheimer’s disease (AD) is characterized by abnormal accumulation of extracellular amyloid-beta (Aβ) and intracellular tau [1], while Parkinson’s disease (PD) is characterized by abnormal intracellular accumulation of alpha-synuclein (αSyn) [2]. Dementia with Lewy bodies (DLB) is characterized by increased Lewy body pathology by disease definition, but also shares pathologies with both AD, including Aβ plaques and tau neurofibrillary tangles, in up to 76% of cases. In contrast, non-dementia PD patients share pathology with AD in fewer cases (7–10%) [3–6]. Although still debated, the consensus emphasizes that the aggregation and toxicity of intermediate toxic seed structures of these pathogenic proteins are considered to be key in disease initiation and progression [7–9].

Naturally occurring autoantibodies (nAbs) are a distinct set of antibodies that recognize self- and non-self-antigens without prior immunization and play a pivotal role in immune clearance of neoepitopes, aggregated and misfolded protein [10]. Although they likely cannot reach the intracellular compartment, they contribute to the engulfment of dying cells and aid in their clearance, while also surveilling the extracellular space, inhibiting the transmission of pathological proteins from cell to cell. They have been found in large amounts in healthy individuals as well in aberrant levels in patients with neurodegenerative diseases such as AD, PD, DLB, and other neurological disorders (summarized in Table S1) [11]. Previous studies have shown alterations in the levels and affinity of nAbs against αSyn, Aβ, and tau in these diseases, suggesting that dysfunction in the immune clearance of pathological proteins may play an considerable role in the development of neurodegenerative diseases [12, 13]. Generally, there is a consistent pattern observed in the levels and functionality of nAbs in neurodegenerative diseases. Early PD and DLB are characterized by increased levels of anti-αSyn nAbs. On the other hand, AD patients, in general, exhibit reduced levels of anti-Aβ nAbs, while no significant differences are observed in anti-tau nAbs (Table S1). Most studies have predominantly focused on IgG nAbs, under the assumption that immune responses following class-switching are of primary importance. However, significant immune functions are also found in the IgM and IgA antibody classes. IgM nAbs, often regarded as the immune system’s “first responders,” can rapidly react to alterations in pathological proteins or result in depletion of inhibitors for protein aggregation [14, 15]. IgA’s on the other hand play a crucial role in mucosal and gut immunity, which has been implicated as a potential mediator in the pathogenesis of neurodegenerative disorders, such as PD and AD [16, 17]. Furthermore, studies evaluating the functionality of nAbs have revealed important insights. PD patients have been found to have reduced affinity of anti-αSyn autoantibodies in both plasma and cerebrospinal fluid (CSF), respectively [18, 19]. This reduction in affinity is also observed in prodromal phases of PD and the atypical parkinsonian disorder multiple system atrophy (MSA) [20]. Similarly, AD patients exhibit reduced affinity for anti-Aβ nAbs [21]. The precise role of nAbs in neurodegenerative disorders, however, remains a subject of ongoing debate and whether these differences in levels, specificity, and efficacy between healthy individuals and those with PD, AD or DLB, suggest that they may contribute to disease onset or progression. However, promising results have been obtained in preclinical animal models, where nAbs have been evaluated in terms of passive immunization (reviewed by [22]). More recently, positive results have been reported in clinical trials for AD using donanemab and lecanemab, both of which target Aβ structures [23, 24].

Here, we evaluated the repertoire of high affinity Immunoglobulin (Ig)G nAbs specific to αSyn, Aβ, and tau in AD, PD, and DLB patients compared to healthy controls. We also investigated the levels of nAbs of different classes (Immunoglobulin (Ig)G, IgM, and IgA). Understanding the connection between nAbs and protein pathology could provide valuable insight into disease mechanisms and identify potential targets for therapeutic treatment.

## Materials and methods

### Demographics

A total of 235 plasma samples were collected from three different biobanks for this study (Table 1 and 2). (1) The samples included 38 PD and 15 DLB patients samples, and 29 control samples from the Bispebjerg Movement Disorder Biobank (BMDB) at the Department of Neurology, Bispebjerg-Frederiksberg Hospital, Copenhagen University Hospital, Denmark (2) 69 AD and 31 DLB patient samples were obtained from the Danish Dementia BioBank (DDBB), Rigshospitalet, Copenhagen University Hospital, Denmark, and (3) 12 PD patients and 41 controls from the research-biobank at the Centre for Neuroscience and Stereology, Bispebjerg-Frederiksberg Hospital, Copenhagen University Hospital, Denmark. Only cases that met the international criteria for probable disease were included in the study [25–28]. The healthy control individuals had no central nervous system conditions, immunological disorders, or ongoing immunomodulatory treatment. All participants provided written consent for inclusion in the biobanks, adhering to the World Medical Association Declaration of Helsinki.

**Table 1:** Demographic and clinical data

	AD (*N* = 69)	PD (*N* = 50)	DLB (*N* = 46)	NC (*N* = 70)	*p* values
Age [years]	70.4 (8.1) [51–89]	68.4 (7.4) [52–84]	72.8 (6.4) [56–88]	71.4 (9.1) [52–90]	0.020*
Sex (M/F)	35/34	24/26	33/13	31/39	0.027**
Age at onset [years]	68.0 (8.6) [48–88]; 81%	61.4 (8.3) [44–78]	69.6 (8.8) [38–87]; 98%	-	0.267^***#***^
MMSE	24.1 (3.9) [12–30]	-	25.8 (4.0) [16–30]; 67%	-	***0.016*** ^**#**^
H&Y	-	2.3 (0.9) [1–5]; 72%	2.4 (0.9) [1–3]; 32%	-	0.461^**#**^
Disease Duration [years]	2.2 (1.2) [0.5-5]; 81%	7.0 (4.1) [0–15]	3.2 (3.3) [0–21]; 98%	-	***< 0.001*** ^***%***^
Biobank	DDBB	BMDB; CNS-lab	DDBB; BMDB	BMDB; CNS-lab	

*: Welch ANOVA. **: chi-squared test. ^**#**^: Mann‒Whitney test. ^%^: Kruskal-wallis test. MMSE: Mini Mental State Examination (MMSE); H&Y: Hoehn & Yahr (7-scale); M: Male; F: Female; DDBB: Danish Dementia BioBank; BMDB: Bispebjerg Movement Disorder Biobank; CNS-lab: Centre for Neuroscience and Stereology. $: PD vs. DLB (collectively), *p* < 0.05

### αSyn/Aβ/tau competition electrochemiluminescence immunoassay (ECLIA)

The affinity of anti-αSyn/Aβ/tau nAbs was assessed based on a competitive antigen-antibody reaction, whereby increasing antigen concentrations in the fluid phase facilitated distinguishable repertoires of high-affinity and low-affinity antibody fractions, previously developed in-house [18]. In this study the assay was adapted and optimized for Aβ and tau. In brief, 96-well mesoscale discovery (MSD) plates were coated overnight at 4 °C with antigens (αSyn: 20 ng/mL (rPeptide, #S-1001), standard small spot MSD plate (MSD, #L45XA); Aβ_1− 42_: 1 µg/mL (rPeptide, #A-1002), standard small spot MSD plate (MSD, #L45XA); tau: 1 ng/mL (rPeptide, #T-1001), high bind plate (MSD, #L15XB)) in ice-cold 0.1 M carbonate buffer, pH 8.5 (Sigma‒Aldrich, #C3041). Next, the plates were blocked for 1 h at 800 rpm (αSyn: PBS + BSA 3% (Sigma‒Aldrich, #05482); Aβ: Intercept™ Blocking Buffer in PBS (LI-COR, #927-90001); tau: ROTI^®^Block1X (Carl Roth, #A151). Meanwhile, plasma samples were diluted (αSyn: 1:200; Aβ/tau: 1:100) in PBS + BSA-0.1% (Sigma‒Aldrich, #05482) and preincubated with the antigen (αSyn: 1000 nM/2 nM/0.2 nM, 0 nM; Aβ: 600 nM/6 nM/0.06 nM/0.0006 nM/0 nM; tau: 100 nM/1 nM/0.01 nM/0 nM) for 1 h before adding onto a newly washed antigen-coated plate (5 times with PBS + 0.05%-Tween-20 (Sigma‒Aldrich, #P7949)) and incubated for 1 h at 800 rpm. After an additional washing step (5 times with PBS + 0.05%-Tween-20 (Sigma‒Aldrich, #P7949)), SULFO-tag goat anti-human (1:10,000; MSD, #R32AJ-1) in PBS + BSA-0.1% (Sigma‒Aldrich, #05482) was added and eventually incubated for 1 h at 800 rpm. Finally, the plate was washed (5 times with PBS + 0.05%-Tween-20) and Read Buffer T (1:2 (MSD, #R92TC)) was added upon reading the plate immediately before the MSD Sector Imager/QuickPlex SQ 120 Reader (MSD, LLC, USA). The percentage of max binding for each sample and pool was calculated as follows:

$$\begin{aligned}&\%\:of\:max\:binding\\&\quad=\frac{({ECLIA}_{sample\:OD}-{ECLIA}_{OD\:at\:1000\:nM\:competitor\:\left(0\%\:binding\right)})}{{ECLIA}_{OD\:at\:0\:nM\:competitor\:\left(100\%\:binding\right)}}\times100.\end{aligned}$$

### IgG, IgM and IgA anti-αSyn/Aβ/tau measurements

Total levels of anti-αSyn/Aβ/tau nAbs were measured by indirect ELISA as previously described [19, 20, 29] with few adjustments. In brief, 96-well polystyrene microtiter plates (Nunc MaxiSorp^®^ flat-bottom) were coated overnight with antigens (αSyn: 5 µg/mL (rPeptide, #S-1001-2); Aβ1–42: 5 µg/mL (rPeptide, A-1002); tau: 0.5 µg/mL (rPeptide, T-1001)) in ice-cold 0.1 M carbonate buffer, pH 8.5 (Sigma‒Aldrich, #C3041). The plates were then emptied and blocked for 2 h at RT with PBS + BSA-3% (Sigma‒Aldrich, #05482) + Tergitol-0.1% (Sigma‒Aldrich #NP40S). Following a subsequent washing cycle of 5 times with PBS + 0.05%-Tween-20 (Sigma‒Aldrich, #P7949), plasma samples were diluted (1:50 for anti-αSyn/Aβ/tau IgA and 1:100 for anti-αSyn/Aβ/tau IgG/IgM) in dilution buffer (PBS + 0.1%BSA + 0.05% + Tween-20) and incubated for 1 h at RT. After another washing cycle (5 times with PBS + 0.05%-Tween-20), secondary HRP-conjugated anti-Ig antibodies (anti-IgG (1:20,000; Abcam, #ab98624), biotin-conjugated anti-IgM (1:5,000; Sigma‒Aldrich, #B1265), and anti-IgA (1:1,000 for αSyn/Aβ; 1:2000 for tau; Thermo Fisher Scientific, #PA1-74395) were diluted in dilution buffer, added to the plates and incubated for 2 h at RT. An additional step was carried out for the biotin-conjugated IgM antibody, with streptavidin–peroxidase (1:10,000; Sigma‒Aldrich, #S5512) for 30 min at RT. Next, the plates were washed once again (5 times with PBS + 0.05%-Tween-20), and tetramethylbenzidine (TMB) Liquid Peroxidase Substrate (Sigma‒Aldrich, #T8665) was added for 30 min in the dark at RT prior to reaction termination by the addition of 0.5 N sulfuric acid (Sigma‒Aldrich, #319570). Finally, the absorbance was measured at 450 nm and 620 nm on a MultiSkan™ FC Microplate Reader (Thermo Fisher Scientific, USA). All data were normalized to positive controls on each individual plate. Positive controls consisted of pooled plasma samples, from controls and patients, added to each plate to account for plate-to-plate variability.

### Statistical analyses

For demographic group comparison, we applied Welch ANOVA followed by the Games-Howell test for multiple comparisons for age since the data was normally distributed but has difference in variances, the chi-squared test for sex, the Mann‒Whitney U test for age at onset, MMSE, Hoehn & Yahr and disease duration. Outliers were removed from analyses using ROUT with false discovery rate (FDR), Q = 1%. Normality was assessed using the D’Agostino Omnibus test. For group comparison, we applied multiple linear regression modeling including covariates age and sex, since small discrepancies between groups were observed, using *ANOVA* from the *car* package [30]. For multiple comparisons, the *glht* and *mcp* functions from the *multicomp* package [31] were applied using Tukey’s range test. Correlations between measured outcomes and clinical data were assessed using Spearman’s rank-order correlation. Spearman’s correlation matrices were constructed using *corrplot* package [32]. Data were analyzed using R v. 3.5.2 [33] and GraphPad Prism 9.4.1 (GraphPad Software Inc., USA).

## Results

### Anti-αSyn/Aβ/tau high-affinity nAbs in AD, DLB and PD patients

**Fig. 1:**
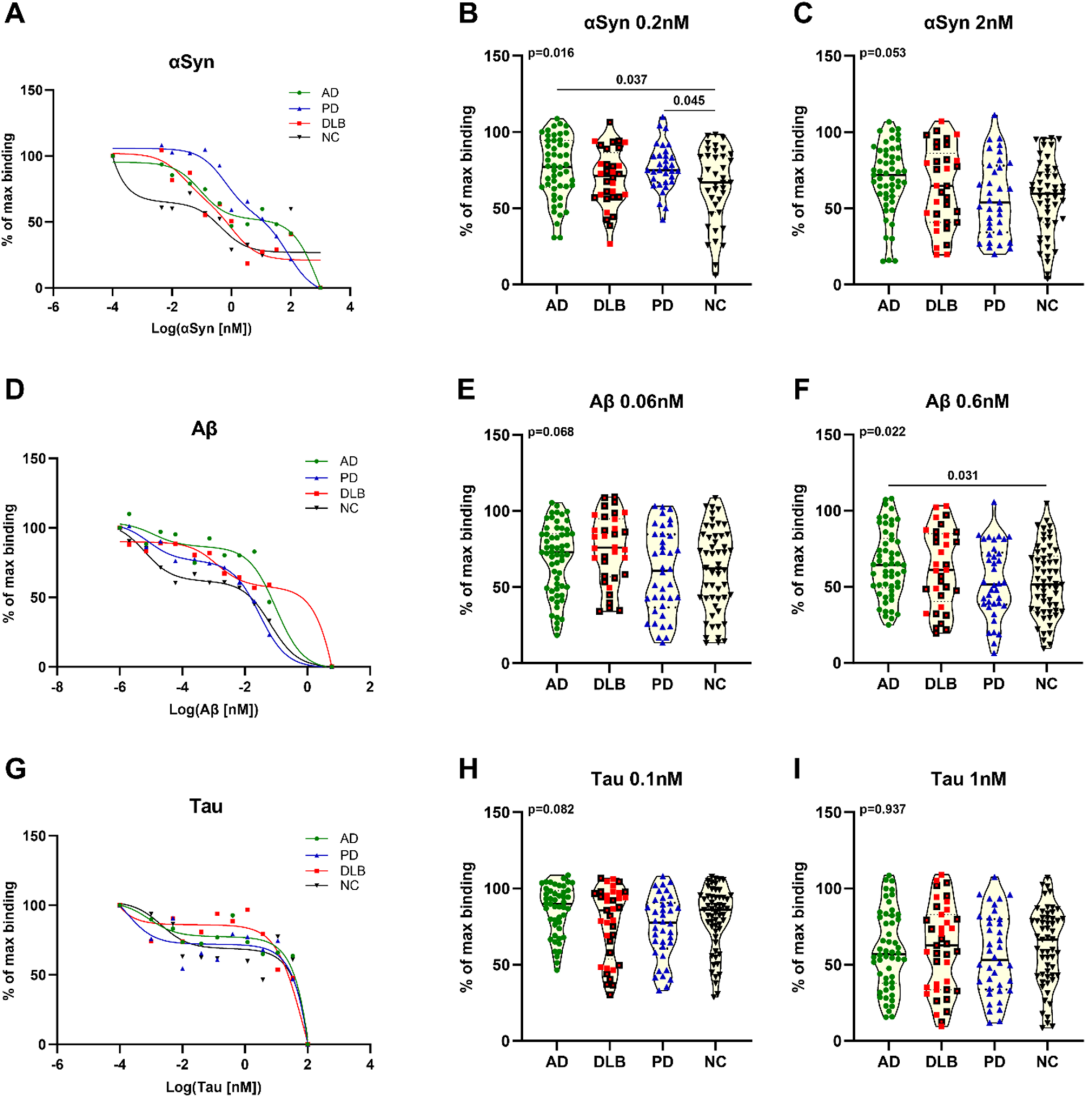
Affinity profiles of anti-αSyn (**A**), -Aβ (**D**) and -tau (**G**) IgG nAbs. Data are presented as two-site inhibition curves of random pooled age- and sex-matched plasma samples (*n* = 10) from normal controls (black triangles and line), AD (green dots and line), PD (blue triangles and line) and DLB (red squares and line). Binding affinities of individual samples of nAbs to αSyn were analyzed in the presence of (**B**) 0.2 nM and (**C**) 2 nM, to Aβ in the presence of (**E**) 0.06 nM and (**F**) 0.6 nM, and to tau in the presence of (**H**) 0.1 nM and (**I**) 1 nM. Data are presented as “% of max binding” in truncated violin plots with median (horizontal line). Group comparisons were performed by applying multiple linear regression models including covariates age and sex and post hoc multiple comparison testing using Tukey’s range test. Statistically significant p-values (< 0.5) are depicted

To assess the functionality of nAbs and their capacity to form stable immunocomplexes across various diseases, we analyzed the binding affinity of anti-αSyn, -Aβ, and -tau IgG nAbs in patients with AD, DLB and PD, as well in control individuals. To perform these analyses, we utilized our well-characterized competition assay with minor adjustments [19, 20, 34]. Based on initial competition curves obtained from a subset of 10 randomly selected age- and sex-matched patients and control individuals (Fig. 1A, D and G), we chose two different conditions to firmly evaluate the high-affinity nAb repertoire. The analysis of individual samples revealed notable differences in the high-affinity repertoire of anti-αSyn and anti-Aβ IgG nAbs. When exposed to 0.2 nM free αSyn, PD patients (*p* = 0.045) and AD patients (*p* = 0.037) (Fig. 1B; Table 3) exhibited a significantly reduced repertoire of high-affinity anti-αSyn IgG compared to controls. Additionally, when exposed to 0.6 nM free Aβ, AD patients only demonstrated significantly lower amounts of high-affinity anti-Aβ IgG compared to controls. No differences in tau affinity reactivity were observed between groups.

**Table 2:** Statistical comparison of nAb affinities and levels for αSyn, Aβ and tau

Antigen	Model statistics			Groups	Sex	Age	DLB-AD	PD-AD	NC-AD	PD-DLB	NC-DLB	NC-PD
***αSyn***	***p value***	***R*** ^***2***^	***F-stat***	***p value***	***p value***	***p value***	p value for multiple comparison*					
2 nM	0.116	0.024	F(5;161) = 1.80	0.053	0.296	0.976						
0.2 nM	***0.033***	0.047	F(5;147) = 2.51	***0.016***	0.317	0.120	0.321	0.997	***0.037***	0.305	0.879	***0.045***
IgG	***1.9E-07***	0.150	F(5,211) = 8.62	***1.2E-07***	0.430	0.686	***0.003***	0.472	***0.023***	***< 0.001***	***< 0.001***	0.640
IgM	***6.1E-07***	0.139	F(5;213) = 8.01	***5.8E-07***	0.286	0.260	0.998	***0.007***	***< 0.001***	***0.045***	***< 0.001***	0.419
IgA	0.334	0.004	F(5;207) = 1.15	0.225	0.168	0.588						
***Aβ***	***p value***	***R*** ^***2***^	***F-stat***	***p value***	***p value***	***p value***	p value for multiple comparison*					
0.6 nM	0.033	0.040	F(5,177) = 2.49	***0.022***	0.450	0.225	0.938	0.098	***0.031***	0.465	0.297	0.998
0.06 nM	0.089	0.025	F(5,177) = 1.95	0.068	0.337	0.915						
IgG	***0.038***	0.030	F(5,224) = 2.41	***0.035***	0.318	0.167	0.640	0.130	***0.032***	0.856	0.637	0.984
IgM	***0.020***	0.037	F(5,222) = 2.76	***0.026***	0.174	0.456	0.616	0.999	0.215	0.686	***0.020***	0.243
IgA	***0.049***	0.026	F(5,227) = 2.26	***0.015***	0.770	0.456	***0.011***	0.333	0.125	0.470	0.611	0.985
***Tau***	***p value***	***R*** ^***2***^	***F-stat***	***p value***	***p value***	***p value***	p value for multiple comparison*					
1 nM	0.817	0.015	F(5,180) = 0.45	0.937	0.267	0.587						
0.1 nM	0.109	0.023	F(5,174) = 1.83	0.082	0.155	0.916						
IgG	***0.033***	0.031	F(5,226) = 2.47	***0.010***	0.533	0.756	0.264	***0.005***	0.213	0.598	0.999	0.389
IgM	***0.020***	0.036	F(5,227) = 2.74	***0.036***	0.057	0.575	0.977	0.091	0.955	0.335	0.826	***0.027***
IgA	0.557	0.005	F(5,225) = 0.79	0.871	0.078	0.891						

*: Multiple comparison modulated for covariables (sex and age)

### Anti-αSyn/Aβ/tau IgG, IgM and IgA nAbs in AD, DLB and PD patients

**Fig. 2:**
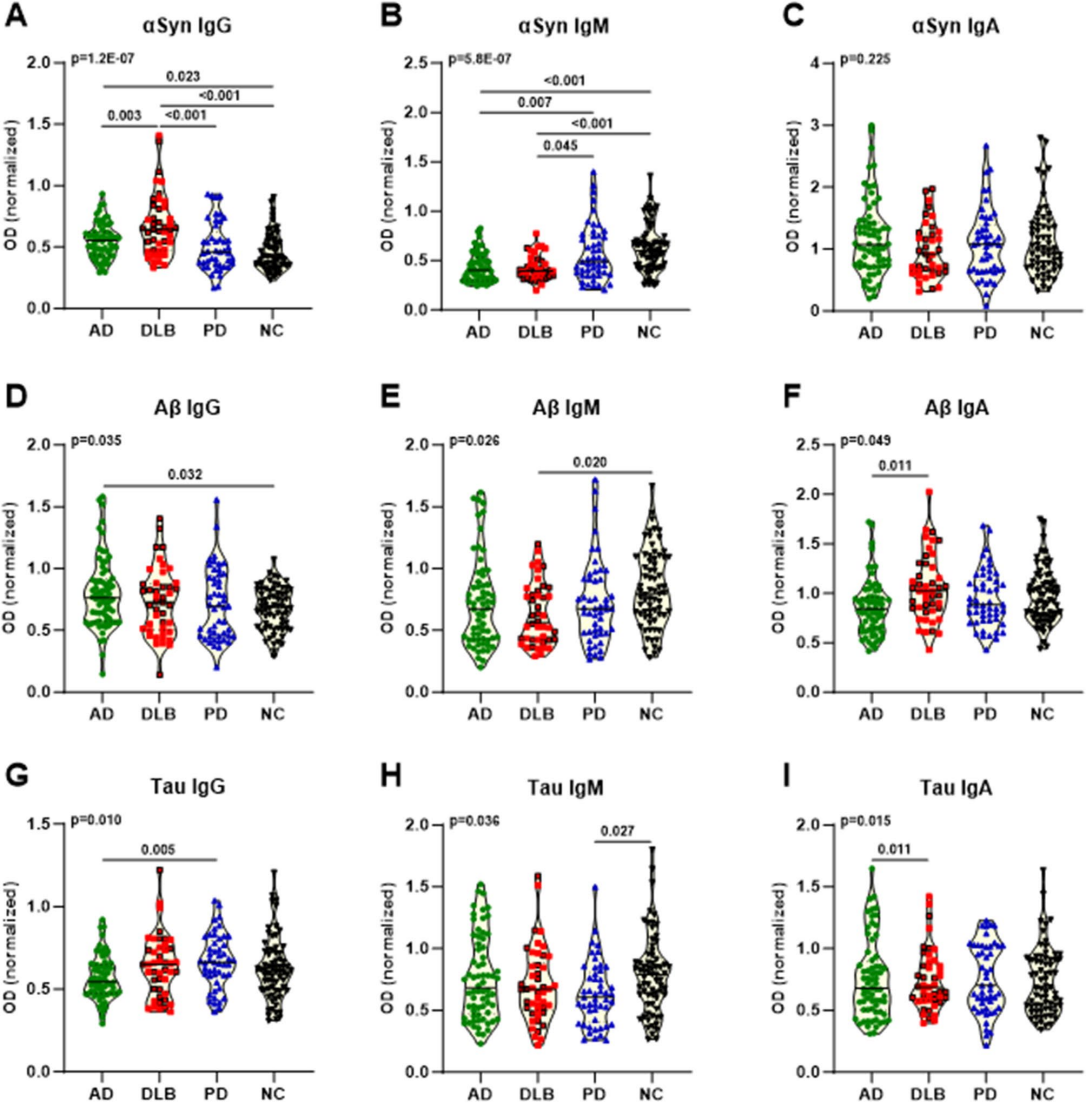
Relative anti-αSyn (**A**-**C**), -Aβ (**D**-**F**) and -tau (**G**-**I**) IgG, IgM and IgA nAb levels in AD, DLB and PD patients as well as controls. Data are presented as normalized optical (normalized to positive controls on each individual plate) densities in truncated violin plots with median (horizontal line). Group comparisons were performed by applying multiple linear regression models including covariates age and sex and post hoc multiple comparison testing using Tukey’s range test. Statistically significant p-values (< 0.5) are depicted

To explore the reactivity of different antibody classes in the immune system, namely, IgG, IgM, and IgA, toward αSyn, Aβ, and tau in patients with AD, DLB, PD, and controls, we conducted indirect ELISA analyses. Exploring the repertoires of anti-αSyn IgG, IgM, and IgA nAbs, we observed that AD and DLB patients exhibited significantly higher levels of anti-αSyn IgG than controls (AD: *p* = 0.023; DLB: *p* < 0.001) (Fig. 2A; Table 3). More significantly, DLB patients exhibited increased levels of anti-αSyn IgG compared to both AD (*p* = 0.003) and PD patients (*p* < 0.001) (Fig. 2A; Table 3). In terms of anti-αSyn IgM, both AD and DLB patients demonstrated reduced levels compared to PD (AD: *p* = 0.007; DLB: *p* = 0.045) and controls (AD: *p* < 0.001; DLB: *p* < 0.001) (Fig. 2B; Table 3). For Aβ, the levels of anti-Aβ IgG were significantly higher in AD patients than in controls (*p* = 0.032) (Fig. 2D; Table 3). In contrast, DLB patients exhibited reduced levels of anti-Aβ IgM compared to controls (*p* = 0.020) (Fig. 2E; Table 3). Furthermore, DLB patients had increased levels of anti-Aβ IgA compared to AD patients (*p* = 0.011) (Fig. 2F; Table 3). Regarding tau, AD patients demonstrated decreased levels of anti-tau IgG compared to PD (*p* < 0.005) patients (Fig. 2G; Table 3), whereas AD patients had increased levels of anti-tau IgA compared to DLB patient (*p* = 0.011). In terms of anti-tau IgM, PD patients had reduced levels compared to controls (*p* = 0.027) (Fig. 2H; Table 3).

### Clinical correlation

Clinical associations were examined to assess the relationship between nAb affinity and levels, and clinical parameters in AD, DLB and PD patients (Table S2, Fig. S1). Interestingly, DLB patients were presented with decreased levels of anti-aSyn IgG levels following disease duration (*r*=-0.457; *p* = 0.002) (Fig. S1A, Table S2) and Hoehn and Yahr (H&Y) staging (*r*=-0.651; *p* = 0.048) (Fig. S1B, Table S2), a commonly used clinical measure of disease severity in diseases with motor impairment, whereas the levels of high affinity anti-aSyn IgG nAbs also decreased during disease duration (*r* = 0.365; *p* = 0.047) (Fig. S1C, Table S2). In PD patients, a significant association was observed between anti-αSyn IgA levels and H&Y staging (*r* = 0.458; *p* = 0.006) (Fig. S1D, Table S2).

### Correlation analysis

**Fig. 3:**
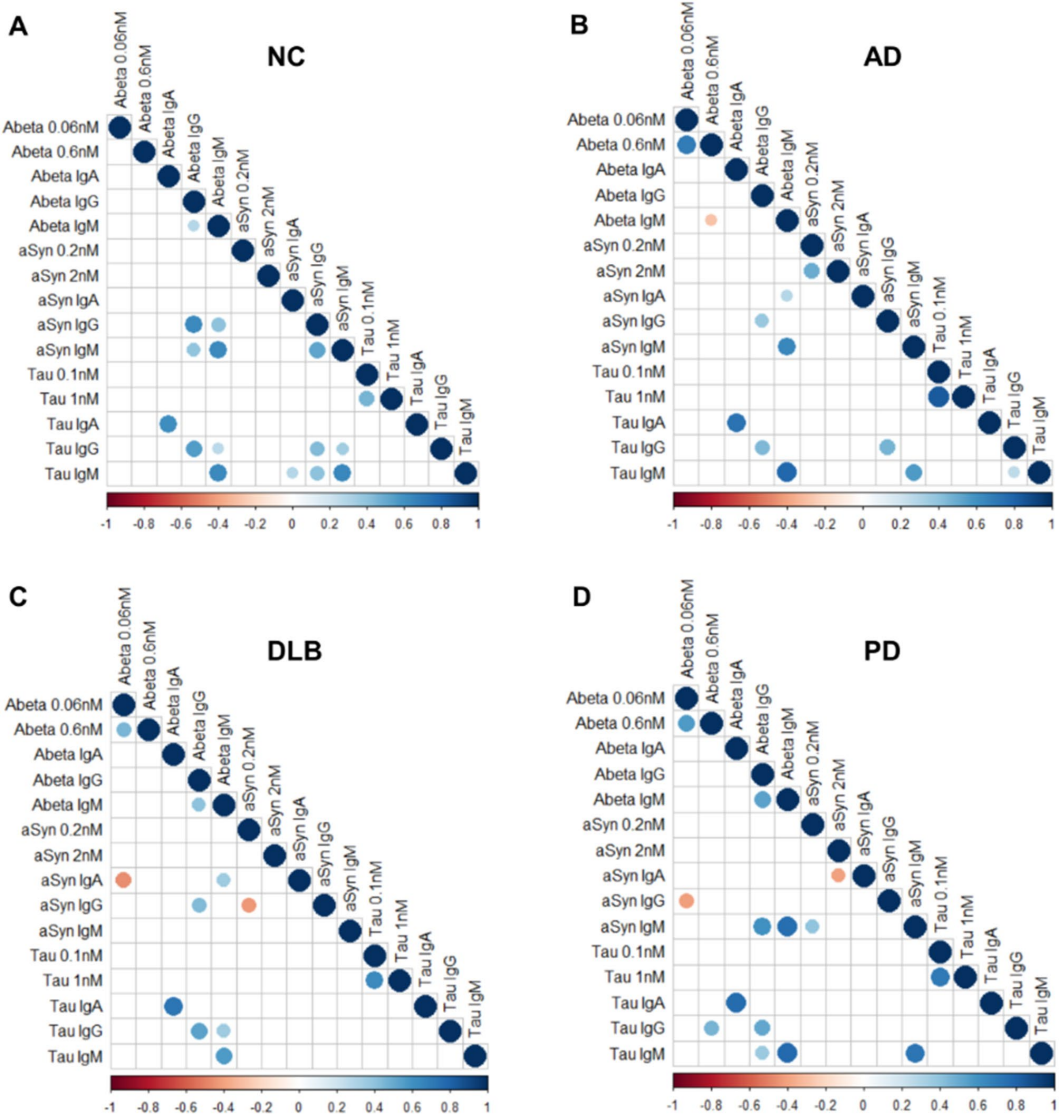
Spearman’s correlation matrix of the anti-αSyn/Aβ/tau affinities and IgGs, IgMs and IgAs levels. Aβ: amyloid-beta, Ig: immunoglobulin, αSyn: alpha-synuclein. Scalebar ranging from Spearman’s *r* = -1 (*red*, negative correlation) to + 1 (*blue*, positive correlation). Only significant correlations are showed. P-values < 0.05 were considered significant

Comprehensive analysis utilizing Spearman’s rank correlation matrices to examine the interrelationships among measured nAbs unveiled multiple significant associations. In the group of healthy controls, positive correlations were observed among the anti-αSyn, anti-Aβ and anti-tau IgG and IgM across all three nAbs, as indicated in Fig. 3A and Table S3. This was with the exception of the anti-Aβ IgG versus anti-tau IgM relationship (*r* = 0.230, *p* = 0.059). Notably, strong correlations persisted across all four examined groups (AD, DLB, PD and controls) for the anti-tau IgA, IgG, IgM and the anti-Aβ IgA, IgG, IgM (Fig. 3A-D, Table S1-4), respectively. Furthermore, a positive correlation was found between anti-Aβ IgM and IgG (*r* = 0.284, *p* = 0.019), and similarly between anti-αSyn IgM and IgG (*r* = 0.525, *p* = 1.2E-05). A positive correlation was also observed between the affinity of anti-tau IgG for two concentrations of free tau (*r* = 0.466, *p* = 4.3E-04). Interestingly, in controls, no correlation was between the two affinity measures for anti-Aβ IgGs (*r*=-0.082, *p* = 0.601), contrasting with the positive correlations observed in AD (*r* = 0.693, *p* = 1.2E-08), DLB (*r* = 0.456, *p* = 0.009) and PD (*r* = 0.567, *p* = 1.7E-04) patients.

In AD patients, while many correlations remained (Fig. 3B, Table S4), there were five exceptions, in addition to the previously described Aβ high-affinity correlation. These exceptions included four positive correlations: between high-affinity anti-αSyn 0.2 nM and anti-αSyn 2 nM (*r* = 0.492, *p* = 4.4E-04), anti-αSyn IgA and anti-Aβ IgM (*r* = 0.289, *p* = 0.024), high affinity anti-tau 0.1 nM and anti-tau 1 nM (*r* = 0.835, *p* = 3.2E-13) and anti-tau IgG versus anti-tau IgM (*r* = 0.259, *p* = 0.034). Additionally, a negative correlation was noted between anti-Aβ IgM and high-affinity anti-Aβ IgGs (*r*=-0.285, *p* = 0.047).

In case of DLB patients compared to healthy controls, eight positive correlations associated with anti-αSyn IgA, IgG, and IgM versus anti-Aβ, and anti-tau IgG, and IgM were eliminated (Fig. 3C, Table S5). Moreover, anti-αSyn IgA and IgG showed negative correlations with high-affinity anti-Aβ IgG (*r*=-0.462, *p* = 0.012) and high-affinity anti-αSyn IgG (*r*=-0.430, *p* = 0.018), respectively. Additionally, the anti-αSyn IgG and anti-αSyn IgM interrelationship was ablated (*r*=-0.117, *p* = 0.497).

In PD patients, compared to healthy controls, there was a notable impact on the correlations involving anti-αSyn and anti-tau IgGs (Fig. 3D, Table S6). Specifically, anti-αSyn IgG exhibited a negative correlation with high-affinity anti-Aβ IgGs (*r*=-0.418, *p* = 0.013), diverging from the previously observed positive correlations with anti-Aβ IgG, anti-tau IgG and IgM in controls, which were no longer present. Furthermore, anti-αSyn IgA was found to be negatively correlated with high-affinity anti-αSyn IgGs (*r*=-0.403, *p* = 0.020). Positive interrelationships were observed between anti-tau IgG and high-affinity anti-Aβ IgGs (*r* = 0.460, *p* = 0.004), anti-tau IgM and anti-Aβ IgGs (*r* = 0.369, *p* = 0.008), and between anti-αSyn IgM and high-affinity anti-αSyn IgGs (*r* = 0.394, *p* = 0.023).

## Discussion

In the explorations of neurodegenerative diseases such as Alzheimer’s disease (AD) and Parkinson’s disease (PD), the role of immune clearance has emerged as a topic of significant interest. This stems from the historical precedent set by the discovery of nAbs against the Aβ protein in AD as early as 2001 [35]. However, even with decades of research, the exact function, and implications of these nAbs remain a subject of debate. Our study offers a fresh perspective by examining the binding affinity of nAbs to essential proteins associated with AD, DLB and PD patients.

By employing our well-characterized competition assay [18–20, 34], we analyzed the high-affinity repertoire of nAbs against αSyn, Aβ, and tau in AD, DLB and PD patients, as well as healthy control subjects. We extended our prior findings in PD to include AD, demonstrating significantly reduced high-affinity anti-αSyn IgG nAbs compared to controls, and further demonstrating reduced high-affinity Aβ nAbs in AD compared to controls. Although the common paradigm separates αSyn pathology into PD and Aβ pathology into AD, the broader landscape of neurodegenerative disorders reveals that between 20 and 40% of all AD cases show pathological αSyn accumulation in the brain [36, 37]. In addition to co-occurrence in pathology, several mechanisms have been proposed for the role of αSyn involvement in AD. αSyn interacts with Aβ and tau, promoting their aggregation and toxicity and contributing to the complexity and severity of neurodegenerative processes [38]. It is likely a key effector in neurotransmitter release and synaptic function, which have been shown to be compromised in AD [39]. Additionally, emerging evidence suggests that αSyn acts as a culprit in neuroinflammatory processes and contributes to activating microglia, as observed both in PD and potentially AD [40, 41]. Although these potential links are intriguing, we can only speculate which processes are present in this study’s AD patients and whether they have αSyn co-pathology, which could explain their reduced functionality of anti-αSyn nAbs.

Earlier research has emphasized the ability of IgG nAbs to regulate inflammation and to facilitate the clearance of neurotoxic aggregates [42]. The presence of disease-specific IgG nAbs targeting pathological proteins such as αSyn, Aβ and tau suggests an intricate interplay between the immune system and the pathogenesis of neurodegenerative disorders [11, 43]. Although the intracellular location of αSyn and tau aggregates makes it unlikely that nAbs penetrate the cell membrane and clear the aggregates intracellularly, they are more likely to scavenge the extracellular space, clearing material transmitting between cells and aiding in the degradation of cellular material after apoptosis. Considering that nAbs play a crucial role in regulating immune clearance mechanisms, any abnormalities in the nAb profile could potentially exacerbate the pathogenesis of neurological conditions. This observation was recently established by the success of two different passive immunization strategies in treating AD. The effectiveness of lecanemab and donanemab in AD patients [23, 24] manifests the importance of functional regulation of key pathological proteins. Passive immunization seems to have the potential to bolster compromised immune system functions in neurodegenerative diseases. To date, no conclusive study of passive immunization in PD patients has proven successful. However, promising secondary outcomes were recently achieved in the AMULET study, a Phase II passive immunization trial in MSA patients. This trial was based on the hypothesis that the treatment would not clear existing aggregates but would instead slow down or halt the spread and seeding of aSyn to other cells [44]. These results, taken together with the lacanemab and donanemab trials in AD, further imply that intravenously administrated antibodies can partially reach the brain, consistent with previous findings showing differences in nAbs in CSF samples from PD and AD, which also correlate with plasma levels [19, 35, 45–48]. Several factors, including the absence of precise antibody candidates, defined pathological hallmarks, and challenges in enrolling patients with varying disease durations or early in the disease onset into the trials, could contribute to this lack of success. Furthermore, the fact that the main pathological processes are different would be the most obvious reason. This contrast may suggest distinct nAb-associated disease mechanisms or pathological responses between AD and PD. Confirming this, recent studies offer seemingly paradoxical perspectives on the role of B cells in disease pathogenesis. For instance, Scott et al. [49]. posited that B cells play a protective role in a PD model, whereas a 2021 study hinted at their pathological role in an AD model [50].

Plasma IgM levels were altered in AD, DLB and PD patients. Interestingly, we observed that AD and DLB patients had reduced anti-αSyn IgM titers compared to PD patients and controls. DLB patients also presented reduced anti-Aβ IgM titers. IgM nAbs bind to disease-specific proteins and influence the aggregation of these proteins [42, 51]. The pentameric structure of IgM endows it with multivalency, allowing it to bind to multiple copies of proteins, such as αSyn, Aβ and tau [52, 53]. This property could facilitate more efficient clearance. IgM’s relatively unspecific yet rapid immune response makes it an essential component of the innate immune system. In neurodegenerative diseases, this could mean that IgM acts as an early responder to neural inflammation or protein aggregation, is secreted quickly, and plays a role in complement activation. Harvesting these properties has been proposed as a passive immunization strategy, with the scFv-Fc format allowing for multimerization into pentameric structures, improving the binding and functionality of the antibodies [54]. On the other hand, reduced antigen-specific IgM nAbs have been observed leading to increased IgG nAbs towards self-antigens [55], possibly explaining the subsequent increased anti-aSyn IgG nAbs in AD and DLB. Further understanding the multifaceted roles of IgM in AD, DLB and PD could offer novel insights into neurodegenerative disease pathogenesis and explore its potential as a therapeutic approach.

Finally, this study is the first to explore the relevance of pathology-related IgA compartments in neurodegenerative diseases. Our data suggest a positive correlation between the Hoehn and Yahr (H & Y) scale and anti-αSyn IgA nAbs in individual plasma samples from PD patients (Fig. S1D). IgAs are primarily known for their role in mucosal immunity, such as lining the gastrointestinal tract, as well as other openings inside the nose and mouth [56]. In addition to their localization at mucosal sites, IgAs are also found in the circulatory system [56]. One of their critical functions is to maintain harmonious homeostasis between the microbiota and the host’s immune response [57]. The role of the gut-brain axis, especially in PD, has attracted increasing interest. One intriguing observation is the identification of αSyn pathology at the gut’s mucosal lining in PD patients [58, 59]. Furthermore, specific infections such as *Helicobacter pylori* have been implicated in PD [60], and urinary tract infections have been associated with the atypical parkinsonian disorder, MSA [61], possibly triggering αSyn misfolding, which can spread to the brain via the vagus nerve [62, 63]. Specifically, related to IgA in the context of the gut-brain axis, recent studies found that the IgA to IgM/IgD ratio was nearly 2-fold increased in PD patients [64]. Moreover, IgA-producing plasma cells are not only present in the meningeal venous sinuses but also associated with increases during aging and after an intestinal barrier breach [65]. B-cell receptor sequencing further identified these cells as originating from the intestine [65]. In the realm of AD, recent research has reported elevated IgA levels in the plasma and brain tissue of APOE-ε4 noncarriers, establishing intracerebral transfer of IgA’s [17]. These and our discoveries indicate a possible connection between gut-specific IgA responses, healthy aging and the onset or progression of PD and related neurodegenerative conditions.

As neurodegenerative pathologies progress and redistribute, distinct changes in nAb function and concentration emerge, with variability across diseases. In AD, Aβ pathology accumulates prior to tau pathology [66], and both occur before clinical symptoms manifest. This sequence implies that nAb-response dynamics may differ between Aβ and tau. Similarly, in PD and DLB, αSyn accumulation is an early event, possibly starting many years prior to disease onset [67, 68], which may explain the heightened anti-αSyn response observed in early and prodromal PD stages [20, 45] and in idiopathic REM sleep behavior disorder (iRBD) patients [49].

The precise interactions among nAb affinity, disease pathology, and immune regulation remain complex and incompletely understood. However, differential responses in nAbs targeting key neurodegenerative proteins suggest intricate interplay between nAb affinity and disease processes. One of the most striking observations is the lack of correlation between the affinity of anti-Aβ IgGs in controls (Fig. 3A) and the strong positive correlations in AD and PD patients (Fig. 3B and D). One hypothesis posits that nAb-producing B cells is pre-existing and slight antigenic pushes, drives generation of IgGs and IgAs by differentiating into plasma cells. This process enables hypermutation and class-switching, as suggested by Reynefeld et al. 2020 [69]. The further persistence of IgG and IgM correlations, particular in controls, suggest that these nAbs may serve protective and regulatory roles, which become disrupted in disease. The reduction in these correlations in patients implies an overall breakdown in the immune system’s ability to coordinate the recognition and removal of misfolded proteins. On the other hand, the disruptions could merely be driven by the chronic presence of Aβ plaques and increased Aβ1–42 levels in the brains of AD patients and αSyn Lewy body aggregates in PD patients, or both in DLB patients and that these high-affinity nAbs are sequestered in the brain, which could contribute to their lower levels in peripheral circulation. However, the non-changes in relation to tau and the absence of correlation between affinity and disease duration, cognitive impairment and motor disability talks against it. Only in DLB patient, a significant decrease was observed in relation to anti-αSyn IgG affinity and levels, suggesting that both affinity and levels are decreased during disease progression (Table S2), suggesting a link between development of αSyn accumulation and anti-αSyn nAbs. This needs to be explored further, in brain and body, and although, we can only speculate at this stage, the breakdown of interrelationships between different nAbs across disease groups support the theory that chronic neuroinflammation and immune dysregulation are shared features across neurodegenerative diseases.

The present study, while insightful, has several limitations. First, its cross-sectional design captures antibody dynamics at a single time point, limiting understanding of their progression over time. Longitudinal studies are needed to clarify these changes, potentially in the prodromal stages. Second, peripheral blood measurements may not fully reflect central nervous system (CNS) pathology, as blood-brain barrier integrity and antibody sequestration in the brain were not directly assessed. The study also focuses on Aβ, tau, and αSyn autoantibodies, potentially overlooking other disease-relevant proteins. The mechanisms behind the observed antibody variations, particularly the role of nAbs in disease progression, remain speculative. Finally, the study did not explore other immune pathways or antibody subclasses that may play critical roles in neurodegeneration. Future research addressing these limitations is necessary for deeper mechanistic understanding.

## Conclusions

The multifaceted nature of neurodegenerative diseases is reflected in the aberrant levels of nAbs and their classes. While the utility of nAbs as diagnostic biomarkers remains a subject of ongoing debate, we argue that their inherent variability among groups and individuals limits their effectiveness in this role. However, their importance in elucidating disease mechanisms should not be underestimated, and they may prove valuable in identifying subgroups within the disease spectrum. Our study provides evidence of a dysfunctional immune system in neurodegenerative diseases, suspected to impair the endogenous clearing mechanism of pathological proteins, namely Aβ, αSyn and tau. This suggests a relationship between disease-specific immunoglobulins and pathogenesis, although the specific nature of this relationship has yet to be clearly defined.

## Electronic supplementary material

Below is the link to the electronic supplementary material.

Supplementary Material 1

## Data Availability

No datasets were generated or analysed during the current study.

## Abbreviations

AD: Alzheimer’s disease
Aβ: Amyloid-beta
PD: Parkinson’s disease
aSyn: Alpha-synuclein
DLB: Dementia with Lewy bodies
nAbs: Naturally occurring autoantibodies
Ig: Immunoglobulin
MSA: Multiple system atrophy
CSF: Cerebrospinal fluid
PDD: Parkinson’s disease dementia
VaD: Vascular dementia
FTD: Frontotemporal dementia
MSA: Multiple system atrophy
PSP: Progressive supranuclear palsy
LRKK2: Leucine-rich repeat kinase 2
bvFTD: Behavior-variant FTD
SCA: Spinocerebellar ataxia
sAD: suspected AD
ADRD: AD-related dementia
NDs: Neurodegenerative disorders
NPH: Normal pressure hydrocephalus
RBD: REM-sleep behavior disorder
ApoE: Apolipoprotein E
E4: Epsilon 4
MCI: Mild cognitive impairment
OND: Other neurological diseases
IC: Inflammatory controls
ALS: Amyotrophic lateral sclerosis
CJD: Creutzfeld-Jakob disease
CBD: Corticobasal degeneration
WE: Wernicke encephalopathy
BMDB: Bispebjerg Movement Disorder Biobank
DDBB: Danish Dementia BioBank
CNS-lab: Centre for Neuroscience and Stereology
ECLIA: Electrochemiluminescence immunoassay
MSD: Mesoscale Discovery
PBS: Phosphate Buffered Saline
BSA: Bovine Serum Albumin
ELISA: Enzyme-linked Immunosorbent assay
TMB: 3,3’, 5,5 tetramethylbenzidine dihydrochloride
FDR: False Discovery Rater
nM: Nanomolar
H&Y: Hoehn & Yahr staging (7-stage)
NC: Non-neurological controls
