# Supplementary material for: Autoantibody profiles in Alzheimer´s, Parkinson´s, and dementia with Lewy bodies: altered IgG affinity and IgG/IgM/IgA responses to alpha-synuclein, amyloid-beta, and tau in disease-specific pathological patterns
Source: J Neuroinflammation. 2024 Dec 3;21:317. doi: 10.1186/s12974-024-03293-3 (PMC11613470; doi:10.1186/s12974-024-03293-3)
Supplement: Supplementary file 1 — Supplementary Material 1 [file 12974_2024_3293_MOESM1_ESM.docx]

**Supplementary material:**

**Title:**

Autoantibody Profiles in Alzheimer´s, Parkinson´s, and Dementia with Lewy Bodies: Altered IgG affinity and IgG/IgM/IgA responses to Alpha-Synuclein, Amyloid-Beta, and Tau in Disease-Specific Pathological Patterns.

**Authors and affiliations:**

Knecht, Luisa^1,2,*^; Dalsbøl, Katrine^1,2,*^; Anja Hviid Simonsen^3^; Pilchner, Falk^4^; Ross, J. Alexander^4^; Winge, Kristian^5^; Salvesen, Lisette^6^; Bech, Sara^6^; Hejl, Anne-Mette^6^; Løkkegaard, Annemette^6^; Hasselbalch, Steen G^3,7^; Dodel, Richard^4^; Aznar, Susana^1,2^; Waldemar, Gunhild^3,7^; Brudek, Tomasz^1,2, #^; Folke, Jonas^1,2,4, #^.

^1^: Centre for Neuroscience and Stereology, Department of Neurology, Bispebjerg and Frederiksberg Hospital, Copenhagen University Hospital, Nielsine Nielsens Vej 6B, DK-2400 Copenhagen NV, Denmark.

^2^: Copenhagen Center for Translational Research, Bispebjerg and Frederiksberg Hospital, Copenhagen University Hospital, Nielsine Nielsens Vej 4B, DK-2400 Copenhagen NV, Denmark.

^3^: Danish Dementia Research Centre, Copenhagen University Hospital - Rigshospitalet, University of Copenhagen, Blegdamsvej 9, DK-2100 Copenhagen Ø, Denmark.

^4^: Chair of Geriatric Medicine and Center for Translational Neuro- and Behavioral Sciences, University Duisburg-Essen, Hufelandstraße 55, DE-45147 Essen, Germany.

^5^: Odense University Hospital, Denmark University of Southern Denmark, Denmark.

^6^: Department of Neurology, Bispebjerg and Frederiksberg Hospital, Copenhagen University Hospital, Nielsine Nielsens Vej 7, DK-2400 Copenhagen NV, Denmark.

^7^: Department of Clinical Medicine, Faculty of Health and Medical Sciences, University of Copenhagen, Blegdamsvej 3B, DK-2100 Copenhagen Ø, Denmark.

**Supplementary figures:** 1

**Supplementary tables:** 6

**Supplementary references:** 40

**Figure 1:** Clinical correlations (aligned with Table S2)


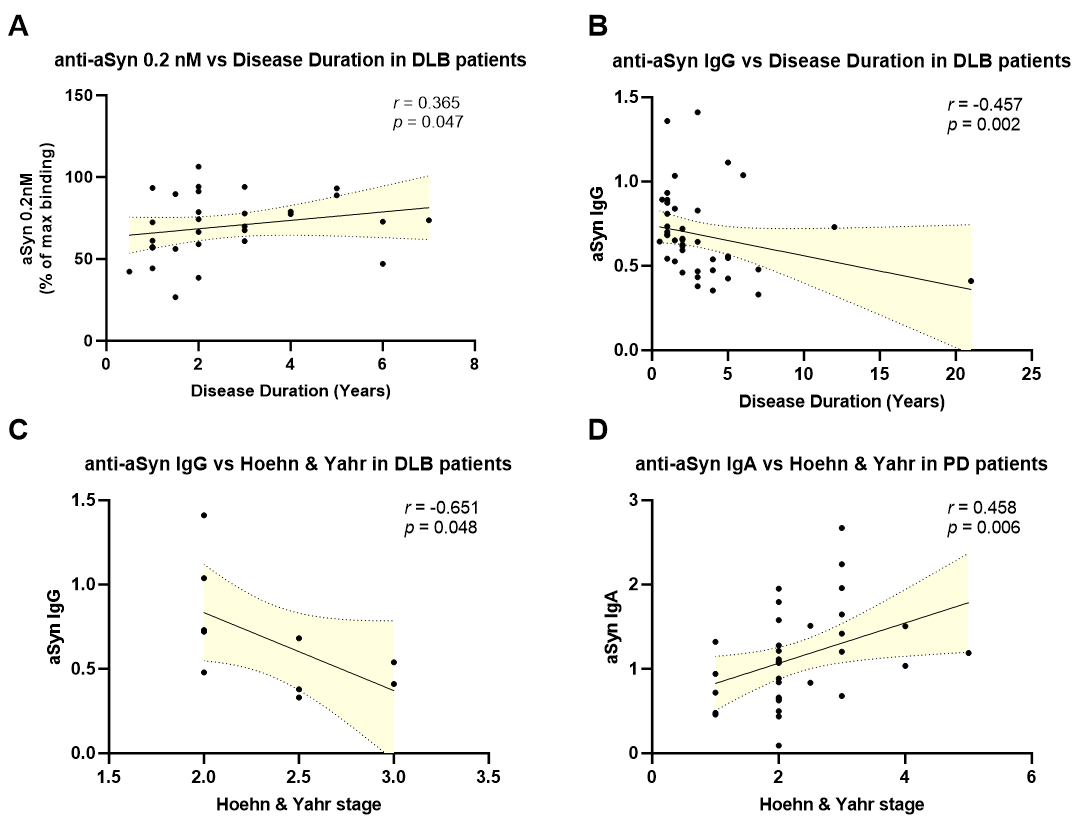


**Figure S1:** Significant clinical association of **A)** anti-αSyn IgG high affinity nAbs in presence of 0.2nM in relation to disease duration in DLB patients, **B)** anti-αSyn IgG levels in relation to disease duration in DLB patients, **C)** anti-αSyn IgG levels in relation to Hoehn and Yahr stage in DLB patients, and **D)** anti-αSyn IgA levels in relation to Hoehn and Yahr stage in PD patients.

**Table S1:** Summary of studies measuring anti-αSyn/Aβ/tau IgG, IgM and IgA nAbs and affinity

| Protein | Biomaterial | Diseases | Findings | Reference |
| --- | --- | --- | --- | --- |
| **αSyn** | Serum | Familial and idiopathic PD | Increased anti-αSyn IgGs in familial PD and total PD compared to controls. No anti-aSyn IgG differences between idiopathic PD and controls. | [1] |
|  | Serum | PD | Increased anti-monomeric-aSyn IgGs in PD compared to controls. Increased anti-oligomeric-αSyn IgGs in early PD compared to controls. | [2] |
|  | Serum + CSF | PDD, DLB, AD, VaD and FTD. | Increased serum anti-aSyn IgGs in PDD/DLB compared to AD, VaD, FTD and controls. No anti-aSyn IgG differences in CSF between groups. | [3] |
|  | Serum | PD (early/late) | Increased anti-αSyn IgGs in PD compared to controls. More profound in early PD. | [4] |
|  | Serum | Depression | No anti-aSyn IgG differences compared to controls. | [5] |
|  | Serum | PD and atypical PD (MSA, PSP and DLB) | No anti-aSyn IgG differences between PD, atypical PD (MSA, PSP and DLB) and controls. | [6] |
|  | Serum | PD and AD | Decreased anti-αSyn IgGs in PD compared to AD and controls. No difference in AD. | [7] |
|  | Plasma | DLB and AD | Increased anti-αSyn IgGs in DLB compared to controls. No differences in AD. | [8] |
|  | Plasma/Serum | PD and LRRK2 mutation carriers | No anti-aSyn IgG differences between PD, LRRK2 mutation carriers and controls. | [9] |
|  | Serum | PD | No anti-aSyn IgG differences compared to controls. | [10] |
|  | Serum | PD, PDD and bvFTD | No anti-aSyn IgG differences between PD and PDD. Unchanged anti-aSyn IgG affinity between PD and PDD. Decreased anti-aSyn IgGs in PD compared to bvFTD. | [11] |
|  | Plasma | PD and MSA | Decreased anti-aSyn IgG affinity in MSA and PD compared to controls. Decreased anti-aSyn-αSyn complexes in MSA and PD compared to controls. | [12] |
|  | Plasma + CSF | PD (early/late) | Increased anti-aSyn IgGs in early PD compared to controls. Increased anti-aSyn IgGs in PD compared to controls. | [13] |
|  | Serum | PD and AD | Increased anti-aSyn IgGs compared to AD and controls. | [14] |
|  | Serum + CSF | PD | No difference of plasma anti-aSyn IgGs compared to controls. Increased CSF anti-aSyn IgGs compared to controls. | [15] |
|  | Plasma | PD and MSA | No anti-aSyn IgG differences compared to controls. Decreased anti-aSyn IgM in PD and MSA. | [16] |
|  | Plasma + CSF | PD and MSA | Decreased anti-aSyn IgG affinity in MSA and PD compared to controls. No differences in CSF anti-aSyn IgGs. Decreased CSF anti-aSyn IgM in MSA and PD compared to controls. No differences in plasma anti-aSyn IgGs. Decreased plasma anti-aSyn IgMs in MSA compared to controls. | [17] |
|  | Serum | PD, tauopathies, sAD, ADRD, MDs (PSP and SCA) and other NDs (NPH). | Decreased anti-aSyn IgGs in PD, MD, AD and NDs compared to controls. Decreased anti-aSyn IgGs in MD compared to PD. | [18] |
|  | Serum | Prodromal PD (pPD) and prodromal MSA (pMSA) | Increased anti-aSyn IgGs in pPD compared to pMSA and NC. Decreased anti-aSyn IgG affinity in pPD (0-8 years prior diagnosis) and pMSA (0-4 years prior diagnosis). | [19] |
|  | Serum | PD, low-risk RBD and high-risk RBD | Increased anti-αSyn-fibril IgGs in high-risk RBD compared to PD, low-risk RBD and controls. No differences in anti-αSyn IgGs. Decreased anti-S129D peptide-aSyn IgG in low-risk RBD compared to PD and controls. | [20] |
|  | Serum | PD and LRKK2-mutation carriers | No anti-αSyn IgGs differences. No anti-aSyn IgG affinity differences measured by surface plasmon resonance. | [21] |
| **Aβ** | Serum + CSF | AD | Decreased plasma and CSF anti-Aβ IgG in AD compared to controls. | [22] |
|  | Serum | AD | No anti-Aβ IgG differences compared to controls. | [23] |
|  | Serum | AD and AD-ApoE-E4 negative | Decreased anti-Aβ IgGs in AD and AD-APOE E4-negative compared to controls. Decreased overall anti-Aβ Igs (IgG, IgA and IgM) in AD compared to controls. | [24] |
|  | Serum + CSF | AD | Increased anti-aggregated-Aβ IgGs in AD compared to controls. Increased anti-unaggregated-Aβ IgGs in AD compared to controls. Only positive in few CSF (3/11). | [25] |
|  | Serum | AD | No anti-Aβ IgG differences compared to controls. | [26] |
|  | Serum | AD, AD-ApoEε4_single_, AD-ApoEε4_double_ | Decreased anti-Aβ IgGs in AD, AD-ApoEε4_single_ and AD-ApoEε4_double_ compared to controls. | [27] |
|  | Serum | AD and MCI | Increased anti-oligomeric-Aβ25-35 IgGs in AD and MCI compared controls. | [28] |
|  | Serum | AD | No anti-monomeric-Aβ IgG differences compared to controls. Decreased anti-oligomeric cross-linked beta-amyloid protein species (CAPS) IgGs in AD compared to controls. | [29] |
|  | Serum | AD | Decreased anti-Aβ IgGs compared to controls. Reduced anti-Aβ affinity in AD compared to controls. | [30] |
|  | Serum + CSF | AD and MCI | Decreased plasma anti-oligomeric-Aβ IgGs with age in controls. Decreased anti-Aβ IgGs in AD compared to MCI. No CSF anti-Aβ IgG differences. | [31] |
|  | Serum + CSF | PDD, DLB, AD, VaD and FTD. | Increased serum anti-Aβ IgGs in PDD and PDD/DLB compared to AD, VaD, FTD and controls. Increased CSF anti-Aβ IgGs in PDD and PDD/DLB, and AD/FTD compared to controls. | [3] |
|  | Serum | AD and MCI | Decreased anti-N-terminal truncated pyroglutamate Aβ (pGluAβ) IgMs in AD compared to controls. | [32] |
|  | Serum | Depression | Decreased compared to NC. | [5] |
|  | Serum | PD | No anti-Aβ IgG differences. | [10] |
|  | Serum | PD, PDD and bvFTD | No difference of anti-Aβ IgG in PDD compared to PD. No difference of anti-Aβ IgGs between PD and bvFTD. | [11] |
|  | Serum + CSF | AD | Increased plasma and CSF anti-Aβ IgGs in AD compared to controls. | [33] |
|  | Serum | AD | No difference of anti-Aβ1-42 IgGs. Decreased anti-Aβ1-15 IgGs in AD compared to controls. Decreased anti-fibrillary-Aβ1-42, -Aβ1-15 and -Aβ16-30 IgGs in AD compared to controls. | [34] |
|  | Plasma | MSA, PD | No anti-Aβ IgG differences. | [16] |
|  | Plasma + CSF | AD | Increased plasma and CSF anti-Aβ1-12 and anti-Aβ7-18 IgGs in AD compared to controls. Decreased plasma and CSF anti-Aβ19-30 and anti-Aβ25-36 IgGs in AD compared to controls. | [35] |
|  | Serum | PD, AD, VaD | Decreased anti-Aβ IgGs in PD compared to AD, VaD and controls. | [36] |
| **Tau** | Serum + CSF | AD, OND | No plasma anti-tau IgG differences between AD and controls. No CSF anti-tau IgG differences between AD and OND. Increased anti-p-tau IgMs in AD compared to controls. No anti-tau IgM differences. | [37] |
|  | Serum + CSF | MS | No serum anti-tau IgG differences. No anti-tau IgG affinity differences. Increased CSF anti-tau IgGs in MS compared to controls. Increased CSF anti-tau avidity in MS compared to controls, more specifically in MS patients without therapy. | [38] |
|  | Serum + CSF | AD, other dementias (PSP, FTD, ALS, CJD, CBD, WE) and IC. | No serum anti-tau IgG differences. Increased CSF anti-tau in IC compared to AD. Decreased serum/CSF ratio in IC compared to AD, other dementias, and controls. | [39] |
|  | Serum + CSF | MS | Increased avidity of nAbs in CSF compared to serum. CSF avidity was increased compared to NC. Serum avidity showed no differences. | [40] |
|  | Serum | PD, PDD and bvFTD | Decreased anti-tau IgG levels in PDD compared to PD. No differences in anti-tau affinity in PDD compared to PD. No difference of anti-tau IgG between PD and bvFTD. | [11] |
|  | Plasma | MSA, PD | No differences in anti-tau IgG and anti-tau IgG compared to controls. | [16] |
|  | Serum | PD, low-risk RBD and high-risk RBD | No differences between groups. | [20] |

Abbreviations: PD: Parkinson’s disease; CSF: cerebrospinal fluid; PDD: Parkinson’s disease dementia; DLB: Dementia with Lewy bodies; AD: Alzheimer’s disease; VaD: Vascular dementia; FTD: Frontotemporal dementia; MSA: Multiple system atrophy; PSP: Progressive supranuclear palsy; LRKK2: Leucine-rich repeat kinase 2; behavior-variant FTD; SCA: Spinocerebellar ataxia; sAD: suspected AD; ADRD: AD-related dementia; NDs: Neurodegenerative disorders; NPH: Normal pressure hydrocephalus; RBD: REM-sleep behavior disorder; ApoE: Apolipoprotein E; E4: Epsilon 4; MCI: Mild cognitive impairment; OND: Other neurological diseases; IC: Inflammatory controls; ALS: Amyotrophic lateral sclerosis; CJD: Creutzfeld-Jakob disease; CBD: Corticobasal degeneration; WE: Wernicke encephalopathy.

**Table S2:** Correlation with clinical data (Spearman’s r)

|  | AD  Disease Duration | | AD  MMSE | | DLB  Disease Duration (All) | | DLB  MMSE  (DDBB) | | DLB  H&Y  (BMDB) | | PD  Disease Duration | | PD  H&Y |  |
| --- | --- | --- | --- | --- | --- | --- | --- | --- | --- | --- | --- | --- | --- | --- |
| **Spearman’s r** | ***r*** | **p-value** | ***r*** | **p-value** | ***r*** | **p-value** | ***r*** | **p-value** | ***r*** | **p-value** | ***r*** | **p-value** | ***r*** | **p-value** |
| Aβ0.06nM | -0.084 | 0.561 | -0.224 | 0.094 | -0.201 | 0.271 | 0.025 | 0.908 | -0.491 | 0.221 | 0.023 | 0.891 | -0.008 | 0.967 |
| Aβ0.6nM | -0.202 | 0.188 | -0.038 | 0.787 | -0.192 | 0.300 | -0.058 | 0.793 | -0.643 | 0.100 | 0.174 | 0.295 | 0.184 | 0.339 |
| Aβ IgA | 0.075 | 0.585 | -0.118 | 0.345 | 0.112 | 0.477 | -0.194 | 0.305 | -0.412 | 0.212 | 0.002 | 0.991 | -0.103 | 0.549 |
| Aβ IgG | 0.138 | 0.311 | 0.200 | 0.104 | -0.231 | 0.141 | 0.088 | 0.637 | -0.206 | 0.548 | -0.158 | 0.279 | -0.169 | 0.325 |
| Aβ IgM | -0.189 | 0.176 | -0.077 | 0.550 | 0.258 | 0.099 | -0.328 | 0.095 | -0.039 | 0.924 | 0.010 | 0.944 | 0.106 | 0.541 |
| αSyn 0.2nM | -0.081 | 0.616 | 0.246 | 0.092 | **0.365** | **0.047** | 0.076 | 0.743 | 0.502 | 0.257 | 0.084 | 0.615 | -0.233 | 0.297 |
| αSyn 2nM | -0.296 | 0.061 | 0.095 | 0.517 | 0.087 | 0.642 | -0.172 | 0.433 | -0.472 | 0.198 | 0.162 | 0.331 | -0.054 | 0.806 |
| αSyn IgA | 0.023 | 0.868 | -0.060 | 0.635 | -0.114 | 0.501 | 0.189 | 0.326 | -0.205 | 0.594 | -0.191 | 0.184 | **0.458** | **0.006** |
| αSyn IgG | 0.014 | 0.920 | -0.003 | 0.984 | **-0.457** | **0.002** | -0.314 | 0.103 | **-0.651** | **0.048** | -0.184 | 0.207 | -0.011 | 0.953 |
| αSyn IgM | -0.175 | 0.226 | -0.027 | 0.841 | 0.065 | 0.703 | 0.006 | 0.978 | 0.077 | 0.895 | -0.165 | 0.253 | -0.057 | 0.744 |
| Tau 0.1nM | 0.232 | 0.173 | -0.168 | 0.259 | 0.168 | 0.327 | 0.004 | 0.984 | -0.149 | 0.730 | -0.072 | 0.696 | -0.017 | 0.931 |
| Tau 1nM | 0.045 | 0.785 | -0.177 | 0.214 | 0.148 | 0.374 | 0.062 | 0.759 | 0.112 | 0.806 | -0.175 | 0.315 | 0.012 | 0.951 |
| Tau IgA | -0.049 | 0.723 | 0.000 | 0.999 | 0.119 | 0.443 | -0.218 | 0.240 | -0.338 | 0.313 | 0.055 | 0.710 | -0.199 | 0.244 |
| Tau IgG | -0.021 | 0.876 | 0.004 | 0.978 | -0.102 | 0.517 | -0.056 | 0.766 | 0.013 | 0.979 | -0.259 | 0.086 | -0.076 | 0.660 |
| Tau IgM | -0.090 | 0.509 | 0.004 | 0.975 | 0.100 | 0.519 | -0.151 | 0.426 | -0.338 | 0.313 | -0.151 | 0.301 | -0.117 | 0.504 |

Abbreviations: AD: Alzheimer’s disease; MMSE: Mini-Mental State Examination; DLB: DLB: Dementia with Lewy bodies; H&Y: Hoehn and Yahr scale; r: Spearman’s rho; Aβ: amyloid-beta; mM: nano molar; Ig: Immunoglobulin; αSyn: alpha-synuclein; Significant results marked in grey.

**Table S3: Correlation matrix for healthy controls**

|  | Aβ 0.06nM | Aβ 0.6nM | Aβ IgA | Aβ IgG | Aβ IgM | αSyn 0.2nM | αSyn 2nM | αSyn IgA | αSyn IgG | αSyn IgM | Tau 0.1nM | Tau 1nM | Tau IgA | Tau IgG |
| --- | --- | --- | --- | --- | --- | --- | --- | --- | --- | --- | --- | --- | --- | --- |
| **Spearman’s r** |  |  |  |  |  |  |  |  |  |  |  |  |  |  |
| Aβ0.6nM | -0.082 |  |  |  |  |  |  |  |  |  |  |  |  |  |
| Aβ IgA | 0.018 | 0.107 |  |  |  |  |  |  |  |  |  |  |  |  |
| Aβ IgG | 0.017 | -0.029 | 0.124 |  |  |  |  |  |  |  |  |  |  |  |
| Aβ IgM | -0.252 | 0.068 | -0.004 | **0.284** |  |  |  |  |  |  |  |  |  |  |
| αSyn 0.2nM | -0.054 | 0.020 | 0.076 | -0.063 | -0.123 |  |  |  |  |  |  |  |  |  |
| αSyn 2nM | -0.158 | 0.070 | -0.096 | -0.085 | -0.015 | 0.238 |  |  |  |  |  |  |  |  |
| αSyn IgA | 0.008 | 0.177 | 0.133 | 0.012 | 0.155 | 0.142 | -0.025 |  |  |  |  |  |  |  |
| αSyn IgG | -0.125 | 0.013 | -0.058 | **0.635** | **0.409** | -0.191 | -0.164 | 0.176 |  |  |  |  |  |  |
| αSyn IgM | -0.234 | -0.131 | -0.126 | **0.394** | **0.636** | 0.200 | -0.035 | 0.122 | **0.525** |  |  |  |  |  |
| Tau 0.1nM | -0.168 | -0.001 | -0.041 | -0.031 | -0.074 | 0.305 | -0.160 | -0.036 | 0.030 | -0.064 |  |  |  |  |
| Tau 1nM | -0.135 | 0.042 | -0.164 | -0.141 | -0.034 | -0.090 | -0.096 | 0.018 | 0.017 | -0.014 | **0.466** |  |  |  |
| Tau IgA | 0.052 | 0.096 | **0.612** | 0.106 | -0.045 | 0.107 | -0.162 | 0.228 | 0.166 | -0.084 | 0.108 | -0.070 |  |  |
| Tau IgG | -0.038 | 0.021 | -0.219 | **0.557** | **0.266** | -0.132 | -0.100 | 0.092 | **0.437** | **0.344** | -0.002 | 0.047 | -0.116 |  |
| Tau IgM | -0.131 | -0.019 | -0.051 | 0.230 | **0.633** | 0.101 | -0.087 | **0.284** | **0.405** | **0.645** | -0.218 | 0.003 | 0.020 | 0.126 |
|  |  |  |  |  |  |  |  |  |  |  |  |  |  |  |
| **P-values** |  |  |  |  |  |  |  |  |  |  |  |  |  |  |
| Aβ0.6nM | 0.601 |  |  |  |  |  |  |  |  |  |  |  |  |  |
| Aβ IgA | 0.902 | 0.423 |  |  |  |  |  |  |  |  |  |  |  |  |
| Aβ IgG | 0.909 | 0.830 | 0.312 |  |  |  |  |  |  |  |  |  |  |  |
| Aβ IgM | 0.071 | 0.612 | 0.973 | **0.019** |  |  |  |  |  |  |  |  |  |  |
| αSyn 0.2nM | 0.774 | 0.914 | 0.646 | 0.711 | 0.455 |  |  |  |  |  |  |  |  |  |
| αSyn 2nM | 0.364 | 0.661 | 0.517 | 0.575 | 0.921 | 0.169 |  |  |  |  |  |  |  |  |
| αSyn IgA | 0.957 | 0.229 | 0.311 | 0.929 | 0.238 | 0.439 | 0.877 |  |  |  |  |  |  |  |
| αSyn IgG | 0.408 | 0.926 | 0.650 | **3.9E-08** | **8.6E-04** | 0.258 | 0.286 | 0.203 |  |  |  |  |  |  |
| αSyn IgM | 0.096 | 0.331 | 0.303 | **9.7E-04** | **4.3E-09** | 0.223 | 0.813 | 0.359 | **1.2E-05** |  |  |  |  |  |
| Tau 0.1nM | 0.294 | 0.994 | 0.764 | 0.818 | 0.587 | 0.095 | 0.331 | 0.805 | 0.836 | 0.635 |  |  |  |  |
| Tau 1nM | 0.399 | 0.778 | 0.224 | 0.304 | 0.801 | 0.624 | 0.557 | 0.904 | 0.903 | 0.919 | **4.3E-04** |  |  |  |
| Tau IgA | 0.716 | 0.478 | **2.3E-08** | 0.392 | 0.711 | 0.523 | 0.277 | 0.080 | 0.197 | 0.495 | 0.430 | 0.607 |  |  |
| Tau IgG | 0.791 | 0.878 | 0.068 | **8.0E-07** | **0.026** | 0.424 | 0.498 | 0.483 | **3.4E-04** | **0.004** | 0.987 | 0.727 | 0.342 |  |
| Tau IgM | 0.354 | 0.890 | 0.678 | 0.059 | **4.1E-09** | 0.542 | 0.555 | **0.028** | **0.001** | **2.2E-09** | 0.104 | 0.984 | 0.870 | 0.297 |

Spearman’s rank correlation matrices showing Spearman’s r and P-values for each correlation. Bold depicts significant correlations. P-values <0.05 were considered significant.

**Table S4: Correlation matrix for AD patients**

|  | Aβ 0.06nM | Aβ 0.6nM | Aβ IgA | Aβ IgG | Aβ IgM | αSyn 0.2nM | αSyn 2nM | αSyn IgA | αSyn IgG | αSyn IgM | Tau 0.1nM | Tau 1nM | Tau IgA | Tau IgG |
| --- | --- | --- | --- | --- | --- | --- | --- | --- | --- | --- | --- | --- | --- | --- |
| **Spearman’s r** |  |  |  |  |  |  |  |  |  |  |  |  |  |  |
| Aβ0.6nM | **0.693** |  |  |  |  |  |  |  |  |  |  |  |  |  |
| Aβ IgA | 0.057 | -0.165 |  |  |  |  |  |  |  |  |  |  |  |  |
| Aβ IgG | -0.110 | 0.002 | -0.049 |  |  |  |  |  |  |  |  |  |  |  |
| Aβ IgM | -0.130 | **-0.285** | 0.086 | 0.078 |  |  |  |  |  |  |  |  |  |  |
| αSyn 0.2nM | 0.184 | 0.050 | -0.026 | 0.258 | 0.134 |  |  |  |  |  |  |  |  |  |
| αSyn 2nM | 0.273 | 0.238 | -0.056 | 0.104 | -0.010 | **0.492** |  |  |  |  |  |  |  |  |
| αSyn IgA | -0.008 | 0.126 | -0.067 | 0.130 | **0.289** | 0.001 | -0.101 |  |  |  |  |  |  |  |
| αSyn IgG | -0.198 | -0.244 | -0.140 | **0.379** | 0.244 | 0.144 | 0.080 | 0.162 |  |  |  |  |  |  |
| αSyn IgM | -0.210 | -0.247 | -0.003 | 0.044 | **0.641** | -0.141 | -0.157 | 0.150 | 0.073 |  |  |  |  |  |
| Tau 0.1nM | -0.041 | -0.022 | -0.001 | 0.067 | -0.023 | 0.120 | 0.081 | -0.145 | -0.119 | 0.031 |  |  |  |  |
| Tau 1nM | 0.103 | 0.209 | -0.011 | 0.135 | 0.082 | 0.175 | 0.174 | 0.069 | -0.037 | -0.060 | **0.835** |  |  |  |
| Tau IgA | -0.028 | -0.115 | **0.731** | 0.103 | 0.072 | -0.086 | -0.114 | -0.063 | -0.009 | -0.160 | -0.025 | -0.004 |  |  |
| Tau IgG | -0.158 | -0.021 | -0.112 | **0.445** | 0.217 | -0.138 | -0.068 | 0.153 | **0.466** | 0.230 | -0.011 | 0.105 | 0.013 |  |
| Tau IgM | -0.158 | -0.239 | -0.059 | 0.057 | **0.789** | 0.051 | -0.110 | 0.173 | 0.236 | **0.561** | 0.130 | 0.109 | -0.045 | **0.259** |
|  |  |  |  |  |  |  |  |  |  |  |  |  |  |  |
| **P-values** |  |  |  |  |  |  |  |  |  |  |  |  |  |  |
| Aβ0.6nM | **1.2E-08** |  |  |  |  |  |  |  |  |  |  |  |  |  |
| Aβ IgA | 0.673 | 0.238 |  |  |  |  |  |  |  |  |  |  |  |  |
| Aβ IgG | 0.407 | 0.987 | 0.689 |  |  |  |  |  |  |  |  |  |  |  |
| Aβ IgM | 0.343 | **0.047** | 0.500 | 0.541 |  |  |  |  |  |  |  |  |  |  |
| αSyn 0.2nM | 0.228 | 0.752 | 0.862 | 0.070 | 0.375 |  |  |  |  |  |  |  |  |  |
| αSyn 2nM | 0.063 | 0.120 | 0.702 | 0.468 | 0.948 | **4.4E-04** |  |  |  |  |  |  |  |  |
| αSyn IgA | 0.953 | 0.377 | 0.595 | 0.299 | **0.024** | 0.994 | 0.492 |  |  |  |  |  |  |  |
| αSyn IgG | 0.144 | 0.085 | 0.271 | **0.002** | 0.061 | 0.339 | 0.591 | 0.209 |  |  |  |  |  |  |
| αSyn IgM | 0.140 | 0.095 | 0.984 | 0.735 | **4.5E-08** | 0.360 | 0.303 | 0.262 | 0.590 |  |  |  |  |  |
| Tau 0.1nM | 0.795 | 0.892 | 0.996 | 0.649 | 0.886 | 0.474 | 0.619 | 0.335 | 0.440 | 0.845 |  |  |  |  |
| Tau 1nM | 0.495 | 0.179 | 0.938 | 0.339 | 0.584 | 0.292 | 0.277 | 0.636 | 0.805 | 0.690 | **3.2E-13** |  |  |  |
| Tau IgA | 0.834 | 0.414 | **2.3E-12** | 0.404 | 0.573 | 0.551 | 0.427 | 0.620 | 0.945 | 0.223 | 0.864 | 0.976 |  |  |
| Tau IgG | 0.237 | 0.882 | 0.367 | **1.5E-04** | 0.088 | 0.343 | 0.640 | 0.225 | **1.0E-04** | 0.077 | 0.939 | 0.462 | 0.916 |  |
| Tau IgM | 0.237 | 0.084 | 0.635 | 0.644 | **1.0E-14** | 0.729 | 0.447 | 0.169 | 0.061 | **2.5E-06** | 0.383 | 0.445 | 0.716 | **0.034** |

Spearman’s rank correlation matrices showing Spearman’s r and P-values for each correlation. Bold depicts significant correlations. P-values <0.05 were considered significant.

**Table S5: Correlation matrix for DLB patients**

|  | Aβ 0.06nM | Aβ 0.6nM | Aβ IgA | Aβ IgG | Aβ IgM | αSyn 0.2nM | αSyn 2nM | αSyn IgA | αSyn IgG | αSyn IgM | Tau 0.1nM | Tau 1nM | Tau IgA | Tau IgG |
| --- | --- | --- | --- | --- | --- | --- | --- | --- | --- | --- | --- | --- | --- | --- |
| **Spearman’s r** |  |  |  |  |  |  |  |  |  |  |  |  |  |  |
| Aβ0.6nM | **0.456** |  |  |  |  |  |  |  |  |  |  |  |  |  |
| Aβ IgA | 0.060 | 0.101 |  |  |  |  |  |  |  |  |  |  |  |  |
| Aβ IgG | -0.185 | -0.275 | 0.147 |  |  |  |  |  |  |  |  |  |  |  |
| Aβ IgM | -0.079 | -0.036 | 0.042 | **0.401** |  |  |  |  |  |  |  |  |  |  |
| αSyn 0.2nM | -0.066 | 0.068 | -0.171 | 0.071 | 0.174 |  |  |  |  |  |  |  |  |  |
| αSyn 2nM | 0.140 | 0.057 | 0.273 | -0.066 | -0.221 | 0.198 |  |  |  |  |  |  |  |  |
| αSyn IgA | **-0.462** | 0.014 | 0.076 | 0.275 | **0.354** | 0.102 | -0.027 |  |  |  |  |  |  |  |
| αSyn IgG | 0.190 | 0.166 | -0.005 | **0.435** | 0.148 | **-0.430** | 0.119 | 0.310 |  |  |  |  |  |  |
| αSyn IgM | -0.065 | -0.055 | -0.120 | -0.003 | 0.297 | 0.247 | -0.176 | -0.014 | -0.117 |  |  |  |  |  |
| Tau 0.1nM | 0.228 | 0.122 | -0.104 | -0.289 | -0.229 | 0.208 | 0.151 | -0.071 | -0.145 | 0.068 |  |  |  |  |
| Tau 1nM | 0.063 | -0.026 | -0.049 | -0.029 | -0.148 | 0.372 | -0.147 | -0.144 | -0.238 | 0.146 | **0.632** |  |  |  |
| Tau IgA | 0.032 | 0.144 | **0.725** | 0.093 | 0.058 | -0.126 | 0.234 | 0.042 | 0.058 | 0.059 | 0.116 | 0.098 |  |  |
| Tau IgG | 0.227 | 0.215 | 0.027 | **0.531** | **0.356** | -0.072 | -0.136 | 0.045 | 0.261 | -0.016 | -0.033 | -0.095 | 0.197 |  |
| Tau IgM | -0.089 | -0.182 | 0.068 | 0.092 | **0.561** | 0.130 | 0.041 | 0.281 | 0.011 | 0.262 | 0.047 | 0.122 | 0.020 | -0.082 |
|  |  |  |  |  |  |  |  |  |  |  |  |  |  |  |
| **P-values** |  |  |  |  |  |  |  |  |  |  |  |  |  |  |
| Aβ0.6nM | **0.009** |  |  |  |  |  |  |  |  |  |  |  |  |  |
| Aβ IgA | 0.742 | 0.589 |  |  |  |  |  |  |  |  |  |  |  |  |
| Aβ IgG | 0.311 | 0.134 | 0.360 |  |  |  |  |  |  |  |  |  |  |  |
| Aβ IgM | 0.668 | 0.848 | 0.793 | **0.010** |  |  |  |  |  |  |  |  |  |  |
| αSyn 0.2nM | 0.770 | 0.771 | 0.357 | 0.715 | 0.358 |  |  |  |  |  |  |  |  |  |
| αSyn 2nM | 0.523 | 0.801 | 0.131 | 0.729 | 0.231 | 0.314 |  |  |  |  |  |  |  |  |
| αSyn IgA | **0.012** | 0.945 | 0.651 | 0.104 | **0.034** | 0.599 | 0.886 |  |  |  |  |  |  |  |
| αSyn IgG | 0.298 | 0.372 | 0.977 | **0.004** | 0.355 | **0.018** | 0.525 | 0.062 |  |  |  |  |  |  |
| αSyn IgM | 0.732 | 0.776 | 0.485 | 0.985 | 0.075 | 0.223 | 0.379 | 0.940 | 0.497 |  |  |  |  |  |
| Tau 0.1nM | 0.253 | 0.553 | 0.545 | 0.092 | 0.193 | 0.307 | 0.462 | 0.697 | 0.398 | 0.724 |  |  |  |  |
| Tau 1nM | 0.748 | 0.894 | 0.774 | 0.869 | 0.388 | 0.056 | 0.466 | 0.423 | 0.157 | 0.435 | **6.0E-05** |  |  |  |
| Tau IgA | 0.864 | 0.441 | **5.6E-08** | 0.563 | 0.719 | 0.507 | 0.205 | 0.803 | 0.717 | 0.734 | 0.508 | 0.564 |  |  |
| Tau IgG | 0.211 | 0.247 | 0.864 | **3.5E-04** | **0.022** | 0.706 | 0.466 | 0.793 | 0.095 | 0.925 | 0.849 | 0.577 | 0.211 |  |
| Tau IgM | 0.623 | 0.318 | 0.664 | 0.564 | **1.1E-04** | 0.485 | 0.823 | 0.088 | 0.944 | 0.117 | 0.786 | 0.465 | 0.898 | 0.602 |

Spearman’s rank correlation matrices showing Spearman’s r and P-values for each correlation. Bold depicts significant correlations. P-values <0.05 were considered significant.

**Table S6: Correlation matrix for PD patients**

|  | Aβ 0.06nM | Aβ 0.6nM | Aβ IgA | Aβ IgG | Aβ IgM | αSyn 0.2nM | αSyn 2nM | αSyn IgA | αSyn IgG | αSyn IgM | Tau 0.1nM | Tau 1nM | Tau IgA | Tau IgG |
| --- | --- | --- | --- | --- | --- | --- | --- | --- | --- | --- | --- | --- | --- | --- |
| **Spearman’s r** |  |  |  |  |  |  |  |  |  |  |  |  |  |  |
| Aβ0.6nM | **0.567** |  |  |  |  |  |  |  |  |  |  |  |  |  |
| Aβ IgA | 0.204 | 0.034 |  |  |  |  |  |  |  |  |  |  |  |  |
| Aβ IgG | -0.171 | 0.245 | 0.104 |  |  |  |  |  |  |  |  |  |  |  |
| Aβ IgM | -0.175 | -0.056 | 0.113 | **0.532** |  |  |  |  |  |  |  |  |  |  |
| αSyn 0.2nM | 0.168 | -0.088 | -0.151 | 0.134 | 0.292 |  |  |  |  |  |  |  |  |  |
| αSyn 2nM | -0.022 | -0.097 | 0.023 | -0.007 | 0.080 | 0.265 |  |  |  |  |  |  |  |  |
| αSyn IgA | -0.016 | 0.020 | -0.092 | -0.190 | 0.136 | -0.095 | **-0.403** |  |  |  |  |  |  |  |
| αSyn IgG | **-0.418** | -0.052 | 0.091 | 0.231 | 0.272 | 0.021 | 0.141 | 0.103 |  |  |  |  |  |  |
| αSyn IgM | -0.038 | 0.002 | 0.163 | **0.608** | **0.759** | **0.394** | 0.156 | -0.154 | 0.181 |  |  |  |  |  |
| Tau 0.1nM | -0.256 | -0.203 | -0.050 | 0.057 | 0.089 | 0.133 | 0.340 | 0.011 | -0.126 | 0.059 |  |  |  |  |
| Tau 1nM | 0.046 | -0.026 | -0.109 | 0.013 | -0.007 | -0.134 | -0.175 | 0.023 | -0.306 | 0.115 | **0.708** |  |  |  |
| Tau IgA | 0.029 | 0.095 | **0.768** | 0.192 | 0.097 | -0.188 | 0.006 | -0.116 | 0.154 | 0.117 | 0.096 | -0.074 |  |  |
| Tau IgG | 0.119 | **0.460** | 0.092 | **0.513** | 0.127 | 0.000 | -0.050 | 0.052 | 0.182 | 0.158 | 0.086 | 0.160 | 0.077 |  |
| Tau IgM | -0.085 | -0.066 | 0.038 | **0.369** | **0.774** | 0.346 | 0.182 | -0.039 | 0.091 | **0.728** | 0.060 | -0.157 | 0.072 | 0.008 |
|  |  |  |  |  |  |  |  |  |  |  |  |  |  |  |
| **P-values** |  |  |  |  |  |  |  |  |  |  |  |  |  |  |
| Aβ0.6nM | **1.7E-04** |  |  |  |  |  |  |  |  |  |  |  |  |  |
| Aβ IgA | 0.212 | 0.836 |  |  |  |  |  |  |  |  |  |  |  |  |
| Aβ IgG | 0.305 | 0.138 | 0.467 |  |  |  |  |  |  |  |  |  |  |  |
| Aβ IgM | 0.286 | 0.734 | 0.427 | **5.9E-05** |  |  |  |  |  |  |  |  |  |  |
| αSyn 0.2nM | 0.423 | 0.677 | 0.401 | 0.464 | 0.099 |  |  |  |  |  |  |  |  |  |
| αSyn 2nM | 0.917 | 0.638 | 0.892 | 0.968 | 0.645 | 0.156 |  |  |  |  |  |  |  |  |
| αSyn IgA | 0.926 | 0.907 | 0.532 | 0.195 | 0.353 | 0.618 | **0.020** |  |  |  |  |  |  |  |
| αSyn IgG | **0.013** | 0.769 | 0.549 | 0.122 | 0.068 | 0.915 | 0.442 | 0.505 |  |  |  |  |  |  |
| αSyn IgM | 0.820 | 0.989 | 0.254 | **2.8E-06** | **1.1E-10** | **0.023** | 0.363 | 0.297 | 0.233 |  |  |  |  |  |
| Tau 0.1nM | 0.189 | 0.300 | 0.761 | 0.731 | 0.591 | 0.526 | 0.076 | 0.947 | 0.464 | 0.723 |  |  |  |  |
| Tau 1nM | 0.816 | 0.897 | 0.511 | 0.940 | 0.968 | 0.515 | 0.374 | 0.891 | 0.078 | 0.493 | **1.9E-06** |  |  |  |
| Tau IgA | 0.861 | 0.571 | **5.0E-11** | 0.178 | 0.500 | 0.303 | 0.974 | 0.434 | 0.307 | 0.420 | 0.562 | 0.659 |  |  |
| Tau IgG | 0.479 | **0.004** | 0.519 | **1.2E-04** | 0.376 | 0.999 | 0.773 | 0.726 | 0.225 | 0.273 | 0.601 | 0.338 | 0.593 |  |
| Tau IgM | 0.613 | 0.695 | 0.792 | **0.008** | **2.8E-11** | 0.052 | 0.294 | 0.792 | 0.553 | **2.1E-09** | 0.722 | 0.345 | 0.621 | 0.958 |

Spearman’s rank correlation matrices showing Spearman’s r and P-values for each correlation. Bold depicts significant correlations. P-values <0.05 were considered significant.

**Supplementary references:**

[1] K.K. Papachroni. N. Ninkina. A. Papapanagiotou. G.M. Hadjigeorgiou. G. Xiromerisiou. A. Papadimitriou. A. Kalofoutis. V.L. Buchman. Autoantibodies to alpha-synuclein in inherited Parkinson’s disease.. J. Neurochem. 101 (2007) 749–756. https://doi.org/10.1111/j.1471-4159.2006.04365.x.

[2] M. a. Gruden. R.D.E. Sewell. K. Yanamandra. T. V. Davidova. V.G. Kucheryanu. E. V. Bocharov. O.R. Bocharova. V. V. Polyschuk. V. V. Sherstnev. L. a. Morozova-Roche. Immunoprotection against toxic biomarkers is retained during Parkinson’s disease progression. J. Neuroimmunol. 233 (2011) 221–227. https://doi.org/10.1016/j.jneuroim.2010.12.001.

[3] W. Maetzler. D. Berg. M. Synofzik. K. Brockmann. J. Godau. A. Melms. T. Gasser. S. Hörnig. M. Langkamp. Autoantibodies against amyloid and glial-derived antigens are increased in serum and cerebrospinal fluid of Lewy body-associated dementias.. J. Alzheimers. Dis. 26 (2011) 171–179. https://doi.org/10.3233/JAD-2011-110221.

[4] K. Yanamandra. M. a. Gruden. V. Casaite. R. Meskys. L. Forsgren. L. a. Morozova-Roche. Alpha-Synuclein Reactive Antibodies As Diagnostic Biomarkers in Blood Sera of Parkinson’S Disease Patients. PLoS One 6 (2011). https://doi.org/10.1371/journal.pone.0018513.

[5] W. Maetzler. M. Langkamp. S. Lerche. J. Godau. K. Brockmann. A. Gaenslen. H. Huber. I. Wurster. R. Niebler. G.W. Eschweiler. D. Berg. Lowered serum amyloid-β1-42 autoantibodies in individuals with lifetime depression.. J. Alzheimers. Dis. 32 (2012) 95–100. https://doi.org/10.3233/JAD-2012-120625.

[6] L.M. Smith. M.C. Schiess. M.P. Coffey. A.C. Klaver. D. a. Loeffler. Alpha-Synuclein and Anti-Alpha-Synuclein Antibodies in Parkinson’s Disease. Atypical Parkinson Syndromes. REM Sleep Behavior Disorder. and Healthy Controls. PLoS One 7 (2012) 1–9. https://doi.org/10.1371/journal.pone.0052285.

[7] D. Besong-Agbo. E. Wolf. F. Jessen. M. Oechsner. E. Hametner. W. Poewe. M. Reindl. W.H. Oertel. C. Noelker. M. Bacher. R. Dodel. Naturally occurring alpha-synuclein autoantibody levels are lower in patients with Parkinson disease. Neurology 80 (2013) 169–175. https://doi.org/10.1212/WNL.0b013e31827b90d1.

[8] N.K.U. Koehler. E. Stransky. M. Shing. S. Gaertner. M. Meyer. B. Schreitmüller. T. Leyhe. C. Laske. W. Maetzler. P. Kahle. M.S. Celej. T.M. Jovin. A.J. Fallgatter. A. Batra. G. Buchkremer. K. Schott. E. Richartz-Salzburger. Altered Serum IgG Levels to α-Synuclein in Dementia with Lewy Bodies and Alzheimer’s Disease. PLoS One 8 (2013) 1–8. https://doi.org/10.1371/journal.pone.0064649.

[9] B. Alvarez-Castelao. A. Gorostidi. J. Ruíz-Martínez. A. López de Munain. J.G. Castaño. Epitope Mapping of Antibodies to Alpha-Synuclein in LRRK2 Mutation Carriers. Idiopathic Parkinson Disease Patients. and Healthy Controls.. Front. Aging Neurosci. 6 (2014) 169. https://doi.org/10.3389/fnagi.2014.00169.

[10] W. Maetzler. A. Apel. M. Langkamp. C. Deuschle. S.S. Dilger. J.G. Stirnkorb. C. Schulte. E. Schleicher. T. Gasser. D. Berg. Comparable autoantibody serum levels against amyloid- and inflammation-associated proteins in Parkinson’s disease patients and controls. PLoS One 9 (2014) 1–6. https://doi.org/10.1371/journal.pone.0088604.

[11] Y. Kronimus. A. Albus. M. Balzer-Geldsetzer. S. Straub. E. Semler. M. Otto. J. Klotsche. R. Dodel. D. Mengel. R. Hilker. S. Baudrexel. E. Kalbe. N. Schmidt. K. Witt. I. Liepelt-Scarfone. S. Gräber. C. Schulte. H.U. Wittchen. O. Riedel. B. Mollenhauer. C. Trenkwalder. T. Klockgether. A. Spottke. U. Wüllner. J.B. Schulz. K. Reetz. I.A. Heber. Naturally occurring Autoantibodies against tau protein are reduced in Parkinson’s disease dementia. PLoS One 11 (2016) 1–15. https://doi.org/10.1371/journal.pone.0164953.

[12] T. Brudek. K. Winge. J. Folke. S. Christensen. K. Fog. B. Pakkenberg. L.Ø. Pedersen. Autoimmune antibody decline in Parkinson’s disease and Multiple System Atrophy; a step towards immunotherapeutic strategies. Mol. Neurodegener. 12 (2017) 44. https://doi.org/10.1186/s13024-017-0187-7.

[13] I. Horvath. I.A. Iashchishyn. L. Forsgren. L.A. Morozova-Roche. Immunochemical Detection of α-Synuclein Autoantibodies in Parkinson’s Disease: Correlation between Plasma and Cerebrospinal Fluid Levels. ACS Chem. Neurosci. 8 (2017) 1170–1176. https://doi.org/10.1021/acschemneuro.7b00063.

[14] A. Shalash. M. Salama. M. Makar. T. Roushdy. H.H. Elrassas. W. Mohamed. M. El-Balkimy. M.A. Donia. Elevated serum α-synuclein autoantibodies in patients with parkinson’s disease relative to Alzheimer’s disease and controls. Front. Neurol. 8 (2017) 1–6. https://doi.org/10.3389/fneur.2017.00720.

[15] R.S. Akhtar. J.P. Licata. K.C. Luk. L.M. Shaw. J.Q. Trojanowski. V.M.-Y. Lee. Measurements of auto-antibodies to α-synuclein in the serum and cerebral spinal fluids of patients with Parkinson’s disease.. J. Neurochem. 145 (2018) 489–503. https://doi.org/10.1111/jnc.14330.

[16] J. Folke. R. Rydbirk. A. Løkkegaard. L. Salvesen. A.-M. Hejl. C. Starhof. S. Bech. K. Winge. S. Christensen. L.Ø. Pedersen. S. Aznar. B. Pakkenberg. T. Brudek. Distinct Autoimmune Anti-α-Synuclein Antibody Patterns in Multiple System Atrophy and Parkinson’s Disease.. Front. Immunol. 10 (2019) 2253. https://doi.org/10.3389/fimmu.2019.02253.

[17] J. Folke. R. Rydbirk. A. Løkkegaard. A.-M. Hejl. K. Winge. C. Starhof. L. Salvesen. L.Ø. Pedersen. S. Aznar. B. Pakkenberg. T. Brudek. Cerebrospinal fluid and plasma distribution of anti-α-synuclein IgMs and IgGs in multiple system atrophy and Parkinson’s disease.. Parkinsonism Relat. Disord. 87 (2021) 98–104. https://doi.org/10.1016/j.parkreldis.2021.05.001.

[18] P. Garg. F. Maass. S.M. Sundaram. B. Mollenhauer. S. Mahajani. C. van Riesen. S. Kügler. M. Bähr. The relevance of synuclein autoantibodies as a biomarker for Parkinson’s disease.. Mol. Cell. Neurosci. 121 (2022) 103746. https://doi.org/10.1016/j.mcn.2022.103746.

[19] J. Folke. E. Bergholt. B. Pakkenberg. S. Aznar. T. Brudek. Alpha-Synuclein Autoimmune Decline in Prodromal Multiple System Atrophy and Parkinson’s Disease. Int. J. Mol. Sci. 23 (2022) 6554. https://doi.org/10.3390/ijms23126554.

[20] K.M. Scott. Y.T. Chong. S. Park. R.S. Wijeyekoon. S. Hayat. R.J. Mathews. Z. Fitzpatrick. P. Tyers. G. Wright. J. Whitby. R.A. Barker. M.T. Hu. C.H. Williams-Gray. M.R. Clatworthy. B lymphocyte responses in Parkinson’s disease and their possible significance in disease progression. Brain Commun. 5 (2023) fcad060. https://doi.org/10.1093/braincomms/fcad060.

[21] A. Albus. Y. Kronimus. M. Burg-Roderfeld. H. van der Wurp. D. Willbold. T. Ziehm. R. Dodel. J.A. Ross. The Avidity of Autoreactive Alpha-Synuclein Antibodies in Leucine-Rich Repeat Kinase 2 Mutation Carriers Is Not Altered Compared to Healthy Controls or Patients with Parkinson’s Disease. Biomolecules 13 (2023). https://doi.org/10.3390/biom13091303.

[22] Y. Du. R. Dodel. H. Hampel. K. Buerger. S. Lin. B. Eastwood. K. Bales. F. Gao. H.J. Moeller. W. Oertel. M. Farlow. S. Paul. Reduced levels of amyloid beta-peptide antibody in Alzheimer disease.. Neurology 57 (2001) 801–805. https://doi.org/10.1212/wnl.57.5.801.

[23] B.T. Hyman. C. Smith. I. Buldyrev. C. Whelan. H. Brown. M.X. Tang. R. Mayeux. Autoantibodies to amyloid-beta and Alzheimer’s disease.. Ann. Neurol. 49 (2001) 808–810. https://doi.org/10.1002/ana.1061.

[24] M.E. Weksler. N. Relkin. R. Turkenich. S. LaRusse. L. Zhou. P. Szabo. Patients with Alzheimer disease have lower levels of serum anti-amyloid peptide antibodies than healthy elderly individuals.. Exp. Gerontol. 37 (2002) 943–948. https://doi.org/10.1016/s0531-5565(02)00029-3.

[25] A. Nath. E. Hall. M. Tuzova. M. Dobbs. M. Jons. C. Anderson. J. Woodward. Z. Guo. W. Fu. R. Kryscio. D. Wekstein. C. Smith. W.R. Markesbery. M.P. Mattson. Autoantibodies to amyloid beta-peptide (Abeta) are increased in Alzheimer’s disease patients and Abeta antibodies can enhance Abeta neurotoxicity: implications for disease pathogenesis and vaccine development.. Neuromolecular Med. 3 (2003) 29–39. https://doi.org/10.1385/nmm:3:1:29.

[26] L. Baril. L. Nicolas. B. Croisile. P. Crozier. C. Hessler. A. Sassolas. J.B. McCormick. E. Trannoy. Immune response to Abeta-peptides in peripheral blood from patients with Alzheimer’s disease and control subjects.. Neurosci. Lett. 355 (2004) 226–230. https://doi.org/10.1016/j.neulet.2003.10.071.

[27] S. Brettschneider. N.G. Morgenthaler. S.J. Teipel. C. Fischer-Schulz. K. Bürger. R. Dodel. Y. Du. H.-J. Möller. A. Bergmann. H. Hampel. Decreased serum amyloid beta(1-42) autoantibody levels in Alzheimer’s disease. determined by a newly developed immuno-precipitation assay with radiolabeled amyloid beta(1-42) peptide.. Biol. Psychiatry 57 (2005) 813–816. https://doi.org/10.1016/j.biopsych.2004.12.008.

[28] M.A. Gruden. T.B. Davidova. M. Malisauskas. R.D.E. Sewell. N.I. Voskresenskaya. K. Wilhelm. E.I. Elistratova. V. V Sherstnev. L.A. Morozova-Roche. Differential neuroimmune markers to the onset of Alzheimer’s disease neurodegeneration and dementia: autoantibodies to Abeta((25-35)) oligomers. S100b and neurotransmitters.. J. Neuroimmunol. 186 (2007) 181–192. https://doi.org/10.1016/j.jneuroim.2007.03.023.

[29] R.D. Moir. K.A. Tseitlin. S. Soscia. B.T. Hyman. M.C. Irizarry. R.E. Tanzi. Autoantibodies to redox-modified oligomeric Abeta are attenuated in the plasma of Alzheimer’s disease patients.. J. Biol. Chem. 280 (2005) 17458–17463. https://doi.org/10.1074/jbc.M414176200.

[30] L. Jianping. Y. Zhibing. Q. Wei. C. Zhikai. X. Jie. L. Jinbiao. Low Avidity and Level of Serum Anti-Aβ Antibodies in Alzheimer Disease. Alzheimer Dis. Assoc. Disord. 20 (2006). https://journals.lww.com/alzheimerjournal/Fulltext/2006/07000/Low_Avidity_and_Level_of_Serum_Anti_A__Antibodies.1.aspx.

[31] M. Britschgi. C.E. Olin. H.T. Johns. Y. Takeda-Uchimura. M.C. LeMieux. K. Rufibach. J. Rajadas. H. Zhang. B. Tomooka. W.H. Robinson. C.M. Clark. A.M. Fagan. D.R. Galasko. D.M. Holtzman. M. Jutel. J.A. Kaye. C.A. Lemere. J. Leszek. G. Li. E.R. Peskind. J.F. Quinn. J.A. Yesavage. J.A. Ghiso. T. Wyss-Coray. Neuroprotective natural antibodies to assemblies of amyloidogenic peptides decrease with normal aging and advancing Alzheimer’s disease.. Proc. Natl. Acad. Sci. U. S. A. 106 (2009) 12145–12150. https://doi.org/10.1073/pnas.0904866106.

[32] A. Marcello. O. Wirths. T. Schneider-Axmann. M. Degerman-Gunnarsson. L. Lannfelt. T.A. Bayer. Reduced levels of IgM autoantibodies against N-truncated pyroglutamate Aβ in plasma of patients with Alzheimer’s disease. Neurobiol. Aging 32 (2011) 1379–1387. https://doi.org/10.1016/j.neurobiolaging.2009.08.011.

[33] M. Maftei. F. Thurm. C. Schnack. H. Tumani. M. Otto. T. Elbert. I.-T. Kolassa. M. Przybylski. M. Manea. C.A.F. von Arnim. Increased levels of antigen-bound β-amyloid autoantibodies in serum and cerebrospinal fluid of Alzheimer’s disease patients.. PLoS One 8 (2013) e68996. https://doi.org/10.1371/journal.pone.0068996.

[34] B.-X. Qu. Y. Gong. C. Moore. M. Fu. D.C. German. L.-Y. Chang. R. Rosenberg. R. Diaz-Arrastia. Beta-amyloid auto-antibodies are reduced in Alzheimer’s disease.. J. Neuroimmunol. 274 (2014) 168–173. https://doi.org/10.1016/j.jneuroim.2014.06.017.

[35] Y.-H. Liu. J. Wang. Q.-X. Li. C.J. Fowler. F. Zeng. J. Deng. Z.-Q. Xu. H.-D. Zhou. J.D. Doecke. V.L. Villemagne. Y.Y. Lim. C.L. Masters. Y.-J. Wang. Association of naturally occurring antibodies to β-amyloid with cognitive decline and cerebral amyloidosis in Alzheimer’s disease.. Sci. Adv. 7 (2021). https://doi.org/10.1126/sciadv.abb0457.

[36] R. Paganelli. A. Paganelli. G. Pawelec. A. Di Iorio. Natural IgG antibodies to β amyloid are decreased in patients with Parkinson’s disease.. Immun. Ageing 20 (2023) 13. https://doi.org/10.1186/s12979-023-00336-w.

[37] H. Rosenmann. Z. Meiner. V. Geylis. O. Abramsky. M. Steinitz. Detection of circulating antibodies against tau protein in its unphosphorylated and in its neurofibrillary tangles-related phosphorylated state in Alzheimer’s disease and healthy subjects.. Neurosci. Lett. 410 (2006) 90–93. https://doi.org/10.1016/j.neulet.2006.01.072.

[38] L. Fialová. A. Bartos. J. Švarcová. I. Malbohan. Increased intrathecal high-avidity anti-tau antibodies in patients with multiple sclerosis. PLoS One 6 (2011) 1–7. https://doi.org/10.1371/journal.pone.0027476.

[39] A. Bartos. L. Fialová. J. Svarcová. D. Ripova. Patients with Alzheimer disease have elevated intrathecal synthesis of antibodies against tau protein and heavy neurofilament.. J. Neuroimmunol. 252 (2012) 100–105. https://doi.org/10.1016/j.jneuroim.2012.08.001.

[40] L. Fialová. J. Švarcová. A. Bartos. I. Malbohan. Avidity of anti-neurocytoskeletal antibodies in cerebrospinal fluid and serum.. Folia Microbiol. (Praha). 57 (2012) 415–419. https://doi.org/10.1007/s12223-012-0105-x.
